# CDK4/6 inhibitors dephosphorylate RNF26 to stabilize TSC1 and increase the sensitivity of ccRCC to mTOR inhibitors

**DOI:** 10.1038/s41416-024-02750-3

**Published:** 2024-06-18

**Authors:** Xinlin Liu, Wei Li, Lu Yi, Jianxi Wang, Wentao Liu, Hongtao Cheng, Shangqing Ren

**Affiliations:** 1 Department of Urology, The Second Xiangya Hospital, Central South University, Changsha, Hunan 410011 China; 2 Uro-Oncology Institute of Central South University, Changsha, Hunan 410011 China; 3 Hunan Engineering Research Center of Smart and Precise Medicine, Changsha, Hunan 410011 China; 4 Department of Urology, The Third Hospital of Changsha, Changsha, Hunan 410011 China; 5 Department of Breast Surgery, Hubei Cancer Hospital, Tongji Medical College, Huazhong University of Science and Technology, Hubei Provincial Clinical Research Center for Breast Cancer, Wuhan Clinical Research Center for Breast Cancer. No.116 Zhuo Daoquan South Road, Wuhan, Hubei 430079 China; 6 Robotic Minimally Invasive Surgery Center, Sichuan Provincial People’s Hospital, School of Medicine, University of Electronic Science and Technology of China, Chengdu, 610072 China

**Keywords:** Renal cell carcinoma, Cancer therapeutic resistance

## Abstract

**Background:**

The combined use of CDK4/6 inhibitors and mTOR inhibitors has achieved some clinical success in ccRCC. Exploring the underlying mechanism of the CDK4/6 pathway in cancer cells and the drug interactions of CDK4/6 inhibitors in combination therapy could help identify new therapeutic strategies for ccRCC. Notably, CDK4/6 inhibitors inactivate the mTOR pathway by increasing the protein levels of TSC1, but the mechanism by which CDK4/6 inhibitors regulate TSC1 is still unclear.

**Methods:**

Mass spectrometry analysis, coimmunoprecipitation analysis, GST pull-down assays, immunofluorescence assays, Western blot analysis and RT‒qPCR analysis were applied to explore the relationships among CDK4, RNF26 and TSC1. Transwell assays, tube formation assays, CCK-8 assays, colony formation assays and xenograft assays were performed to examine the biological role of RNF26 in renal cancer cells.TCGA-KIRC dataset analysis and RT‒qPCR analysis were used to examine the pathways affected by RNF26 silencing.

**Results:**

CDK4/6 inhibitors stabilized TSC1 in cancer cells. We showed that CDK4 enhances the interaction between TSC1 and RNF26 and that RNF26 activates the mTOR signaling pathway in ccRCC, contributes to ccRCC progression and angiogenesis, and promotes tumorigenesis. We then found that RNF26 functions as an E3 ligase of TSC1 to regulate CDK4-induced TSC1. This finding suggested that RNF26 promotes ccRCC progression and angiogenesis to some extent by negatively regulating TSC1.

**Conclusion:**

Our results revealed a novel CDK4/RNF26/TSC1 axis that regulates the anticancer efficacy of CDK4/6 inhibitors and mTOR inhibitors in ccRCC.

## Introduction

Renal cancer is one of the three most common malignant tumors of the urinary system, and it ranks among the top 10 in terms of incidence among human malignant tumors worldwide [1]. Clear cell renal cell carcinoma (ccRCC) is the main histological type of renal cell carcinoma (RCC), accounting for approximately 75% of all RCC cases, and is one of the main types leading to RCC-related death [2]. Most cases of ccRCC are in an advanced stage when they are first diagnosed, and nearly 30% of cases present with metastasis at first diagnosis [3]. In recent years, the clinical application of antiangiogenic drugs has significantly improved the prognosis of patients with advanced ccRCC [4]. However, long-term use of antiangiogenic drugs reduces the sensitivity of ccRCC patients to these drugs [5]. Therefore, there is an urgent need to find new treatment strategies to address the resistance of ccRCC to antiangiogenic drugs.

Agents specifically targeting cyclin-dependent kinases 4 or 6 (cyclin-dependent kinases 4/6, CDK4/6) (palbociclib, ribociclib, etc.) have a high affinity for CDK4/6, which can lead to G1-phase cell cycle arrest and tumor growth inhibition [6, 7]. CDK4/6 inhibitors have been approved for the clinical treatment of breast cancer, and it has been found that they have synergistic effects with estrogen antagonists in breast cancer cell lines [8–10]. In addition, CDK4/6 inhibitors have been clinically tested in various tumors, such as liver cancer, lung cancer, and prostate cancer [11–13]. Moreover, the combined administration of CDK4/6 inhibitors with other anticancer inhibitors, such as tyrosine kinase inhibitors (TKIs) or mammalian target of rapamycin (mTOR) inhibitors, has been tested in solid tumors [14–16]. Both TKIs and mTOR inhibitors are first-line or second-line drugs for the clinical treatment of ccRCC [17]. The combined use of CDK4/6 inhibitors and mTOR inhibitors has achieved some clinical success in treating ccRCC [18]. Therefore, exploring the underlying mechanism of the CDK4/6 pathway in cancer cells and the interactions of CDK4/6 inhibitors in combination therapy could help identify new therapeutic strategies for ccRCC.

As an important cell cycle signal transduction molecule, CDK4/6 can combine with three subtypes of the cyclin D family to form the cyclin D-CDK4/6 complex, which promotes the phosphorylation of the Rb tumor suppressor factor and releases the transcription factor E2F to activate related genes, inducing the transition of the cell cycle from G1 phase to S phase [19]. This process promotes the transcription of cell cycle gene products, forming a positive feedback loop to initiate DNA synthesis beyond the limits of cell cycle progression [19]. Aberrant hyperactivation of the cyclin D-CDK4/6-Rb signaling pathway promotes the progression of solid tumors by controlling the cell cycle and cell proliferation [20]. Selective CDK4/6 inhibitors can inactivate cyclin D-CDK4/6 by binding to CDK4/6-ATP, thereby inhibiting the phosphorylation of Rb and arresting the cell cycle in the G1 phase [21]. In addition, CDK4/6 could enhance the progression of malignancies independent of Rb. For instance, cyclin D-CDK4/6 activates mTORC1 and regulates cell growth by binding and phosphorylating tuberous sclerosis 2 (TSC2) at Ser1217 and Ser1452 [22]. TSC2 normally inhibits mTORC1; however, CDK4 or CDK6 phosphorylation of TSC2 releases mTOR from TSC2-mediated inhibition, similar to the mechanism by which Rb is phosphorylated by CDK4 or CDK6 to release E2F [22]. TSC2 is also a binding protein of cyclin D, and co-overexpression of cyclin D and CDK4 or CDK6 in human embryonic kidney fibroblasts promoted the phosphorylation of the TSC1-TSC2 complex and decreased the level of the TSC1-TSC2 complex to impair the function of the TSC1-TSC2 complex and activate the mTOR signaling pathway [23]. After the TSC1-TSC2 complex is inhibited, mTOR signaling is further activated to promote cell growth by promoting the phosphorylation of the mTOR downstream targets 4E-binding protein 1 (4EBP1) and S6 kinase (S6K) [23]. Notably, treatment with CDK4/6 inhibitors increases the protein levels of TSC1 and TSC2 [23]. CDK4/6 inhibitors increase the protein levels of TSC2 through non-RB pathways [23], but the mechanism by which CDK4/6 inhibitors regulate TSC1 is still unclear. As an important member of the TSC1-TSC2 complex, TSC1 has significant research implications because of its interaction with CDK4/6. While previous studies have indicated that CDK4/6 inhibitors can affect the mTOR signaling pathway by regulating TSC2, reports on the regulatory role of CDK4/6 on TSC1 are relatively limited. By delving more deeply into the interactions between TSC1 and CDK4, we aimed to elucidate the significance of this mechanism in the development of ccRCC, providing a more profound understanding for the development of novel therapeutic strategies in the future.

Ring finger protein 26 (RNF26) is an E3 ubiquitin ligase belonging to the RING finger domain protein family. RNF26 functions by conjugating ubiquitin molecules to target proteins, thereby marking them for degradation or regulating their functions [24]. The role of RNF26 in urological tumors has rarely been addressed. Our previous studies indicated that RNF26 is upregulated in bladder cancer, leading to tumor growth by inducing p57 instability [25]. Additionally, in ccRCC, we confirmed that RNF26 mediates the degradation of CBX7, thereby promoting the proliferation of renal cancer cells [26]. We believe that the biological role of RNF26 in renal cancer remains incompletely understood and warrants further investigation.

In this study, we explored the mechanism by which CDK4/6 inhibitors increase the protein expression of TSC1 in ccRCC. We demonstrated that CDK4/6 inhibitors stabilized TSC1 in cancer cells. Treatment with CDK4/6 inhibitors led to the dephosphorylation of RNF26 and decreased the interaction between RNF26 and TSC1 to prevent the degradation of TSC1. Therefore, RNF26 is recognized as an ideal candidate for modulating the sensitivity of ccRCC to CDK4/6 inhibitors and mTOR inhibitors.

## Material and methods

### Cell lines and cell culture

Renal clear cell carcinoma cell lines A498 (#CL-0254, Procell Life Science&Technology, Wuhan, China) and HUVEC (#CL-0122, Procell Life Science&Technology, Wuhan, China) were purchased from Procell Life Science&Technology and identified by short tandem repeat (STR) profiling. 786-O (SC0154) cells were purchased from the Yuchicell Biology Technology (Shanghai, China) and identified by STR profiling. The basic medium for 786-O cells was 1640 culture medium, for A498 cells was MEM culture medium, and for HUVEC was Ham’s F-12K culture medium. All of them were incubated at 37 °C in 5% CO_2_ and cultured in different kinds of basic medium added with 10% fetal bovine serum (FBS) (Newzerum, Christchurch) and 1% Penicillin streptomycin mixture (P/S) (#R20016, Biosharp, Guangzhou, China).

### Chemicals and reagent

CDK4/6 inhibitors:Palbociclib (PD-0332991) HCl (#S1116), Proteasome inhibitors: MG132 (#S2619), mTOR inhibitors: Everolimus (#S1120), Bafilomycin A1 (BafA1), (#S1413) were purchased from Selleck (Shanghai, China). The shRNAs were purchased from GeneCopoeia (USA). The siRNAs were obtained from RiboBio (Guangzhou, China). The sequences of siRNA and shRNA are provided in the Table S1.

### Immunohistochemistry (IHC)

Immunohistochemistry was performed with primary antibodies against RNF26 (#16802-1-AP, Proteintech, Wuhan, China 1:1000 dilution), TSC1 (#29906-1-AP, Proteintech, Wuhan, China, 1:1000 dilution), CDK4 (#11026-1-AP, Proteintech, Wuhan, China, 1:1000 dilution), p-S6K1 (Thr389) (#28735-1-AP, Proteintech, 1:1000 dilution), and VEGFA (#19003-1-AP, Proteintech, 1:1000 dilution) in the tissue microarray slides (#U081ki01, Bioaitech, Xian, China). The degree of binding was evaluated by multiplying the intensity score by the proportion of cells stained. The staining intensity scores were as follows: 0 (no staining), 1 (light yellow = weak staining), 2 (yellow brown = moderate staining), 3 (brown = strong staining).

### Quantitative real-time PCR analysis

Total RNA was extracted from cells using TRIzol reagent (Termo Fisher Scientifc, USA). Reverse transcription was performed using Evo M-MLV reverse transcription premixed kit (#AG11728, Accurate Biology, Hunan, China), and qRT-PCR was subsequently performed using Evo M-MLV one-step RT-qPCR kit (#AG11732, Accurate Biology, Hunan, China), following the manufacturer’s instructions. β-actin was used as an internal control. The primer sequences (Bgi-write, Beijing, China) for RT‒PCR are provided in Table S2.

### Western blotting assay

The protein was extracted with protein lysate (#G2002, Servicebio, Wuhan, China) added with protease inhibitor (#G2006, Servicebio, Wuhan, China) and phosphatase inhibitor (#G2007, Servicebio, Wuhan, China), and quantified using BCA Protein Detection Kit (#P0011, Beyotime, Shanghai, China). Lysate proteins were separated by sodium dodecyl sulfate-polyacrylamide gel electrophoresis (SDS-PAGE), transferred to a 0.45 μm polyvinylidene fluoride membrane (Millipore, Billerica). After sealing with 5% skim milk, the membranes were incubated with corresponding primary antibody overnight at 4°C and secondary antibody for 1 h at room temperature. Protein signals were visualized using ECL detection reagent (Termo Fisher Scientifc, USA) and ChemiDoc XRS (Bio-Rad Laboratories, USA). The primary antibodies used were as follows: RNF26 (#16802-1-AP, Proteintech, 1:1000 dilution), TSC1 (#29906-1-AP, Proteintech, 1:2000 dilution), CDK4 (#11026-1-AP, Proteintech, 1:2000 dilution), S6K1 (#14485-1-AP, Proteintech, 1:4000 dilution), p-S6K1 (Thr389) (#28735-1-AP, Proteintech, 1:4000 dilution), VEGFA (#19003-1-AP, Proteintech, 1:2000 dilution), p-RB1(phospho S795) (#ab47474, Abcam, 1:500 dilution), GAPDH (#60004-1-Ig, Proteintech, 1:50000 dilution), and pRB1 S249/T252 (#701059, Thermo fisher scientific, 1:1000 dilution).

### Transwell-invasion assays

Transwell-invasion assays were performed using 8-μm pore BioCoat Matrigel Invasion chambers (Corning, USA). The cells were starved with serum-free medium for 24 h. After spreading the matrix glue in the upper chamber of the chamber, the cells were digested into single cells with trypsin and re-suspended in the serum-free medium. Adding cells to the upper chamber according to the density of 6 × 10^4^ cells per well, and the medium containing 10%FBS was added to the lower chamber. After 24 h of incubation, the cells were stained with 0.1% crystal violet for 30 min, and the unmigrated cells in the upper chamber were carefully removed with cotton swabs. Four visual fields were randomly selected under the microscope to count the cells passing through the membrane.

### Colony formation assays

The cells were digested into single cells with trypsin and suspended in complete culture medium. The cell suspension was added to a 6-well plate at a density of 100, 200, 500 per well and cultured at 37 °C and 5% CO_2_. Replacing the culture medium in a week and abandoning the medium in two weeks. The cells were washed twice with PBS and fixed with paraformaldehyde for 20 min, and then stained with crystal violet for 30 min. Washing off the dye solution and counting the number of clones. Clone formation rate = (numbers of clones / number of inoculated cells) × 100%.

### Angiogenesis assay in vitro

Matrix gel, sterile 24-well plate, pipette tips and other instruments used in the experiment were placed at 4 °C overnight the day before the experiment. The matrix glue was added to the 24-well plate at 250 μL per well with precooled pipette tips. The plate was incubated at 37 °C for 1 h until the matrix glue solidified. When the confluency of HUVEC reached 80%, they were digested by trypsin and resuspended with different tumor-conditioned supernatant. The cells were inoculated on the board containing matrix glue at 1 × 10^4^ per well, and three replicates were made in each group. Cells were cultured and monitored for tube formation up to 48 h. Four visual fields were randomly selected for each well, and the number of tubes were counted and photographed.

### Nude mouse xenograft assay

All animal experiments were examined and approved by the Institutional Animal Care and Use Committee (IACUC) of the Second Xiangya Hospital, Central South University (animal license number 20230474). Animal experiments are consistent with the guidelines for the National Institutes of Health Guide for the Care and Use of Laboratory Animals (NIH Publications No. 8023, revised 1978). BALB/C-nu/nu mice (6 weeks old) were purchased from SJA Laboratory Animal Company (Changsha, China). Since the sex of the mice had no effect on the results of the study, we chose half the males and half the females for the experiment. The mice were placed under standard conditions with a light / dark cycle of 12 h. They had easy access to food and water. Specific shRNA and shControl were used to transfect cells for 72 h, and puromycin was used to screen shRNA positive cells for 48 h. The cells were digested with trypsin and centrifuged by 1050 RPM for 5 min. After adding proper amount of PBS and fully mixing, the cell suspension was stored on ice and injected subcutaneously on the left side of the back of mice (5 × 10^6^ cells per mice) as soon as possible (n = 5 mice per group). The length and width of the tumor were measured with vernier caliper every 2 days, and the tumor volume was calculated according to the formula (L × W^2^)/2. The mice were euthanized and the tumors were removed, photographed and weighed.

### Immunofluorescence staining

786-O cells were inoculated on glass coverslips. Being fixed with 4% paraformaldehyde for 15 min, the cells were subsequently incubated with anti-RNF26 (#16802-1-AP, Proteintech, 1:200 dilution) or anti-TSC1 (#29906-1-AP, Proteintech, 1:250 dilution) antibody at 4 °C overnight, and washed several times with PBS. Then the cells were incubated with suitable fluorophore-conjugated secondary antibody for 1 h at room temperature in the dark. The slides were washed repeatedly with PBS and incubated with DAPI (#C1002, Beyotime, Shanghai, China) for 5 min. Images were captured using a fluorescence microscope.

### Statistical analysis

The results were analyzed by using GraphPadPrism5 software. The sample size (N) of each statistical analysis is provided in the figure legends. The values between the two groups were compared by unpaired bilateral student’s t test, and multiple comparisons were made by one-way analysis of variance (ANOVA), followed by Tukey’s post-hoc test. In all cases, *P* < 0.05 was considered statistically significant, and the significance of differences was indicated as follows: *P < 0.05; **P < 0.01; and ***P < 0.001.

## Results

### CDK4/6 inhibitors prevent the proteasomal degradation of TSC1 in renal cancer cells

**Fig. 1:**
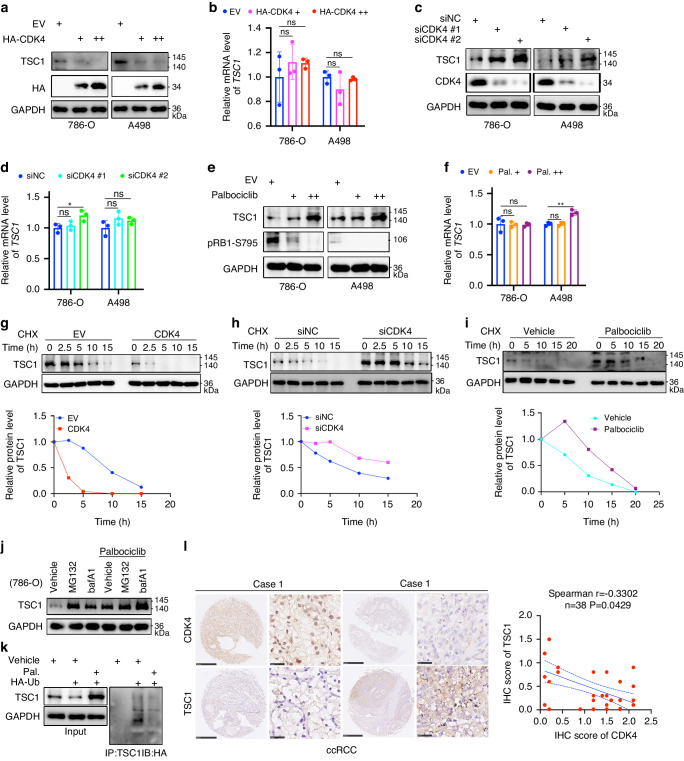
CDK4/6 inhibitors prevent the proteasomal degradation of TSC1 in renal cancer cells. 786-O and A498 cells were transfected with EV, HA-CDK4 (1 ng, +), and HA-CDK4 (5 ng, ++) for 24 h. Cells were harvested for western blotting (**a**) and RT-qPCR (**b**) assay. Data are presented as the mean ± SEM of three replicates. ns, not significant. **c**, **d** 786-O and A498 cells were transfected with indicated siRNAs for 48 h. Cells were harvested for western blotting (**c**) and RT-qPCR (**d**) assay. Data are presented as the mean ± SEM of three replicates. ns, not significant; *, P < 0.05. **e**, **f** 786-O and A498 cells were treated with vehicle, Palbociclib (1 μM, +), or (10 μM, ++) for 24 hours. Cells were harvested for western blotting (**e**) and RT-qPCR (**f**) assay. Data are presented as the mean ± SEM of three replicates. ns, not significant; **, P < 0.01. **g–i** 786-O cells were transfected with the indicated plasmids or siRNAs for 48 h, or treated with Palbociclib (5 μM) for 24 h. Cells were treated with CHX, and cells were collected for western blotting analysis at different time points. **j** 786-O cells were treated with indicated chemicals for 48 h and subjected to western blotting analysis. **k** 786-O cells were treated with indicated chemicals for 48 h and subjected to immunoprecipitation and western blotting analysis. **l** IHC staining of the tissue microarray of ccRCC (n = 38) with CDK4 or TSC1 antibodies.

We sought to investigate how a CDK4/6 inhibitor modulates the expression of TSC1 in cancer cells. First, ectopically overexpressed CDK4 decreased the protein levels of TSC1 in 786-O and A498 cells (Fig. 1a). However, overexpression of CDK4 did not affect the mRNA levels of TSC1 in these cancer cells (Fig. 1b). In contrast, we demonstrated that knockdown of CDK4 significantly increased the protein level of TSC1 (Fig. 1c) but slightly changed the mRNA level of TSC1 in 786-O and A498 cells (Fig. 1d). Then, 786-O and A498 cells were treated with different doses of palbociclib for 24 hours (Fig. 1e). We showed that palbociclib treatment increased the protein but not the mRNA levels of TSC1 in a dose-dependent manner (Figs. 1e, f). Similarly, overexpression of CDK4 shortened the protein half-life of TSC1, but knockdown of CDK4 or palbociclib treatment prolonged the protein half-life of TSC1 in 786-O cells (Fig. 1g–i). Moreover, we showed that the proteasome inhibitor MG132 and lysosome inhibitor BafA1 increased the protein level of TSC1 in 786-O cells (Fig. 1j), and the effect of MG132 on TSC1 after palbociclib treatment was also obvious compared to that of BafA1 (Fig. 1j). Moreover, palbociclib treatment suppressed the polyubiquitination of TSC1 in 786-O cells (Fig. 1k). In addition, IHC staining with CDK4 and TSC1 antibodies revealed a negative correlation between CDK4 and TSC1 expression in the ccRCC tissue microarray (Spearman r = –0.3302, *P* = 0.0429, *n* = 38) (Fig. 1l). Therefore, these data indicate that CDK4/6 inhibitors are involved in regulating the protein stability of TSC1 in cancer cells.

### CDK4 strengthens the binding of RNF26 and TSC1 in renal cancer cells

**Fig. 2:**
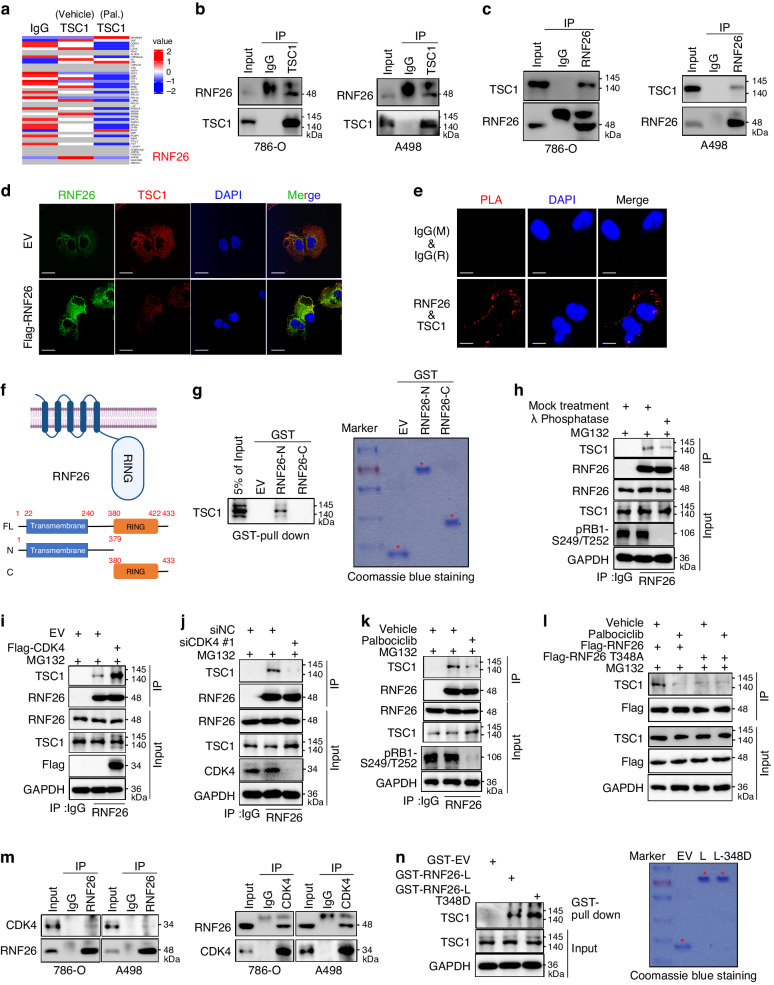
CDK4 strengthens the binding of RNF26 and TSC1 in renal cancer cells. **a** 786-O cells were treated with or without Palbociclib (5 μM) for 24 h. Cells were harvested for immunoprecipitation and mass spectrometry analysis by using the IgG or TSC1 antibodies. **b**, **c** 786-O and A498 cells were harvested for immunoprecipitation by using the TSC1 or RNF26 antibodies. **d** 786-O cells were transfected with indicated plasmids for 48 h. Cells were harvested for immunofluorescence staining by using the RNF26 or TSC1 antibodies. **e** Proximity Ligation Assay (PLA) was performed in the 786-O cells by using the indicated antibodies. **f** A model depicting the domain of RNF26. **g** GST-pull down assay was performed by using the recombinant protein of RNF26. **h** 786-O cells were treated with indicated chemicals for 24 h. Cells were harvested for immunoprecipitation and western blotting assay. **i, j** 786-O cells were treated with indicated plasmids or siRNAs for 48 h. Cells were harvested for immunoprecipitation and western blotting assay. **k** 786-O cells were treated with indicated chemicals for 24 h. Cells were harvested for immunoprecipitation and western blotting assay. **l** 786-O cells were treated with indicated plasmids or chemicals for 48 h. Cells were harvested for immunoprecipitation and western blotting assay. **m** 786-O and A498 cells were harvested for immunoprecipitation by using the CDK4 or RNF26 antibodies. **n** GST-pull down assay was performed by using the recombinant protein of RNF26.

To explore the underlying mechanism by which CDK4/6 inhibitors modulate the stability of TSC1 in ccRCC, 786-O cells treated with or without palbociclib were subjected to mass spectrometry with IgG or TSC1 antibodies (Fig. 2a). We standardized the results using the Z score method, where darker red represents stronger binding to TSC1 and larger positive values, and conversely, darker blue represents weaker binding to TSC1 and smaller negative values. Notably, we found that RING finger protein 26 (RNF26), which was previously identified as an E3 ligase that degrades p57 and CBX7 [25, 26], could be a potential binding partner of TSC1 (Fig. 2a). Mass spectrometry also demonstrated that treatment with palbociclib decreased the binding of TSC1 and RNF26 (Fig. 2a). Next, we explored whether RNF26 is the key mediator that regulates the stability of TSC1 induced by palbociclib.

First, a coimmunoprecipitation (co-IP) assay revealed that RNF26 interacted with TSC1 in 786-O and A498 cells (Fig. 2b, c). Immunofluorescence and proximity ligation assays also revealed that TSC1 colocalized with and bound to RNF26 in 786-O cells (Fig. 2d, e). Moreover, we constructed two GST-tagged plasmids that were translated into two recombinant proteins, namely, GST-RNF26-N (1-379 aa) and GST-RNF26-C (380-433 aa), according to the structure of RNF26 (Fig. 2f). A GST pull-down assay demonstrated that TSC1 bound to the N-terminus of RNF26 (Fig. 2g).

RNF26 may affect the expression of TSC1 in 786-O cells. RNF26 overexpression experiments verified that the expression of TSC1 decreased (Fig. 2d), and we speculated that RNF26 might be involved in regulating the protein level of TSC1. Considering these findings and the finding from mass spectrometry that palbociclib treatment decreased the interaction between RNF26 and TSC1 (Fig. 2a), we studied whether CDK4/6-mediated phosphorylation influenced binding between TSC1 and RNF26. First, we showed that phosphatase treatment decreased the binding of TSC1 to RNF26 in 786-O cells (Fig. 2h). Overexpression of CDK4 increased the interaction between TSC1 and RNF26 (Fig. 2i), but CDK4 depletion or palbociclib treatment reduced this interaction (Fig. 2j, k). Interestingly, a site (threonine, T) in the amino acid sequence of RNF26 could be phosphorylated by CDK (Supplementary Fig. 1a). We constructed an RNF26 T348A (threonine to alanine) mutant to mimic the dephosphorylation of RNF26 at the T348 site (Fig. 2l). We showed that the RNF26 T348A mutant bound less TSC1 than did wild-type RNF26, and palbociclib suppressed the interaction between RNF26 T348A and TSC1 in 786-O cells (Fig. 2l). Furthermore, co-IP assays showed that CDK4 interacted with RNF26 in 786-O and A498 cells (Fig. 2m). A GST pull-down assay also demonstrated that RNF26-T348D (threonine to aspartate), which mimics the phosphorylated state, bound more TSC1 than did wild-type RNF26 (Fig. 2n). Thus, the above data suggest that CDK4 phosphorylates RNF26 at the T348 site to enhance the interaction between TSC1 and RNF26.

### RNF26 promotes the progression and angiogenesis of ccRCC

**Fig. 3:**
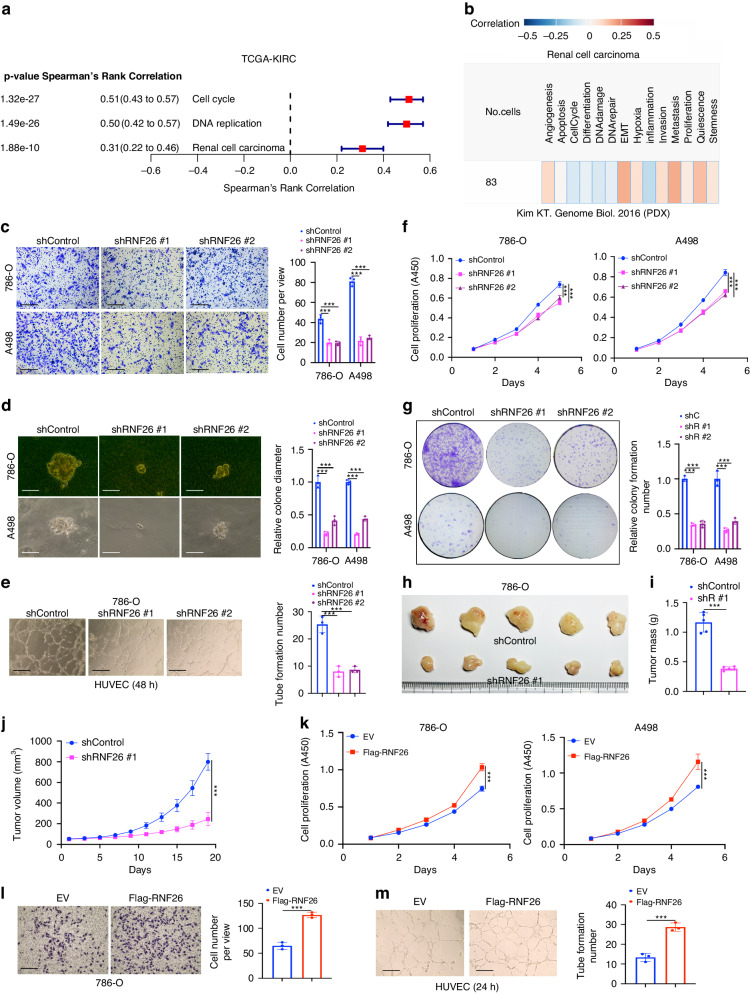
RNF26 promotes the progression and angiogenesis of ccRCC. **a** Analysis of the TCGA-KIRC dataset after sub-dividing the RNF26 into high or low expression group. **b** CancerSEA web tool (http://biocc.hrbmu.edu.cn/CancerSEA/) was used to analyze the biological function of RNF26 in the RCC. **c**–**j** 786-O and A498 cells were infected with indicates shRNAs for 72 h. Cells were harvested for transwell, colony formation, tube formation, CCK-8 and nude mouse xenograft assay. Data are presented as the mean ± SEM of three replicates for in vitro assay and fix replicates for in vivo assay. ***, P < 0.001. **k** 786-O and A498 cells were transfected with indicates plasmids for 48 h. Cells were harvested for CCK-8, transwell and tube formation assay. Data are presented as the mean ± SEM of three replicates. ***, P < 0.001. **l**, **m** 786-O cells were transfected with indicated plasmids for 24 h. Cells were harvested for transwell and tube formation assay. Data are presented as the mean ± SEM of three replicates. ***, P < 0.001.

We previously mentioned that RNF26 acts as an E3 ligase to degrade CBX7 in ccRCC [26], but the cancer-related role of RNF26 in ccRCC is still unclear. We showed that RNF26 was positively correlated with the cell cycle, DNA replication and RCC-related pathways through analysis of the TCGA-KIRC dataset (Fig. 3a). CancerSEA web tool analysis revealed that RNF26 was positively associated with the biological processes of epithelial–mesenchymal transition (EMT), invasion, stemness, proliferation and angiogenesis in ccRCC cells (Fig. 3b). Specifically, we demonstrated that knockdown of RNF26 significantly decreased cell migration, proliferation and stemness in 786-O and A498 cells (Fig. 3c–g, Supplementary Fig. 2a). The nude mouse study also revealed that inhibition of RNF26 reduced tumor growth in vivo (Fig. 3h–j). In contrast, RNF26 overexpression promoted proliferation, migration and angiogenesis in vitro (Fig. 3k–m, Supplementary Fig. 2b). These results indicate that RNF26 contributes to ccRCC progression and angiogenesis.

### RNF26 activates the mTOR signaling pathway in ccRCC cells

**Fig. 4:**
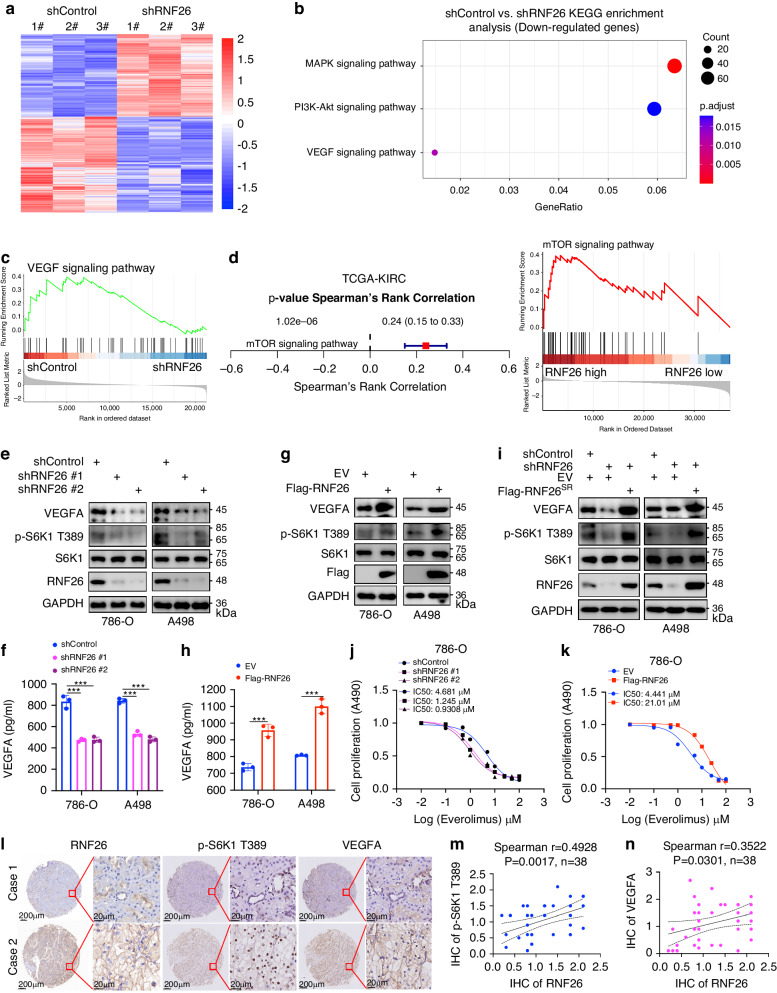
RNF26 activates the mTOR signaling pathway in ccRCC cells. **a–c** A498 cells were infected with indicated shRNAs for 72 h. Cells were harvested and subjected to RNA-seq analysis (**a**). KEGG and GSEA analysis were performed. **d** GSEA analysis of TCGA-KIRC was performed after sub-dividing the RNF26 into high or low expression group. **e**, **f** 786-O and A498 cells were infected with indicates shRNAs for 72 h. Cells were harvested for western blotting analysis (**e**) and ELISA assay (**f**). Data are presented as the mean ± SEM of three replicates. ***, P < 0.001. **g**, **h** 786-O and A498 cells were transfected with indicates plasmids for 48 h. Cells were harvested for western blotting analysis (**g**) and ELISA assay (**h**). Data are presented as the mean ± SEM of three replicates. ***, P < 0.001. **i** 786-O and A498 cells were transfected with indicated constructs for 72 h. Cells were harvested for western blotting analysis. SR, shRNA resistant. **j**, **k** 786-O cells were transfected with indicated constructs for 72 h. Cells were harvested and treated with a serial dose of Everolimus for 24 h to measure the IC50 values of Everolimus. **l–n** IHC staining of the tissue microarray of ccRCC (n = 38) with RNF26, p-S6K1 T389 or VEGFA antibodies.

We continued to study the underlying mechanism of RNF26 in the tumorigenesis of ccRCC. RNF26 was knocked down in A498 cells, which were subjected to RNA-seq analysis (Fig. 4a). KEGG enrichment analysis indicated that RNF26 knockdown inactivated the MAPK, PI3K-AKT, and VEGF signaling pathways (Fig. 4b, c). Analysis of the TCGA-KIRC dataset indicated that RNF26 is positively correlated with the mTOR signaling pathway (Fig. 4d). Given that the PI3K-AKT signaling pathway activates the mTOR signaling pathway and that the mTOR signaling pathway is responsible for the activation of the VEGF signaling pathway and is closely associated with the angiogenesis and progression of tumors, we explored whether RNF26 regulates the activation of the mTOR pathway in ccRCC cells. According to RNA-seq of RNF26, RNF26 knockdown decreased the mRNA level of VEGFA, which is regulated by the mTOR signaling pathway [27] (Fig. 4a). Similarly, we revealed that RNF26 knockdown decreased VEGFA expression and S6K1 T389 phosphorylation in both 786-O and A498 cells (Fig. 4e, f and Supplementary Fig. 2c). In contrast, overexpression of RNF26 promoted VEGFA expression and S6K1 T389 phosphorylation in ccRCC cells (Fig. 4h, g and Supplementary Fig. 2d). The rescue experiment also showed that RNF26 regulated the levels of VEGFA and p-S6K1 (T389) in ccRCC cells (Fig. 4i and Supplementary Fig. 2e). Moreover, we found that depletion of RNF26 decreased the IC50 of the mTOR inhibitor everolimus, but overexpression of RNF26 increased the IC50 of everolimus (Fig. 4j, k). In addition, the IHC staining assay indicated that the protein level of RNF26 was positively correlated with the levels of VEGFA (Spearman r = 0.4928, P = 0.0017, n = 38) and p-S6K1 T389 (Spearman r = 0.3522, P = 0.0301, n = 38) in the ccRCC tissue microarray (Fig. 4l–n). Together, these data indicate that RNF26 plays an important role in activating the mTOR signaling pathway in ccRCC.

### mTOR signaling is critical for determining the role of RNF26 in promoting ccRCC tumorigenesis

**Fig. 5:**
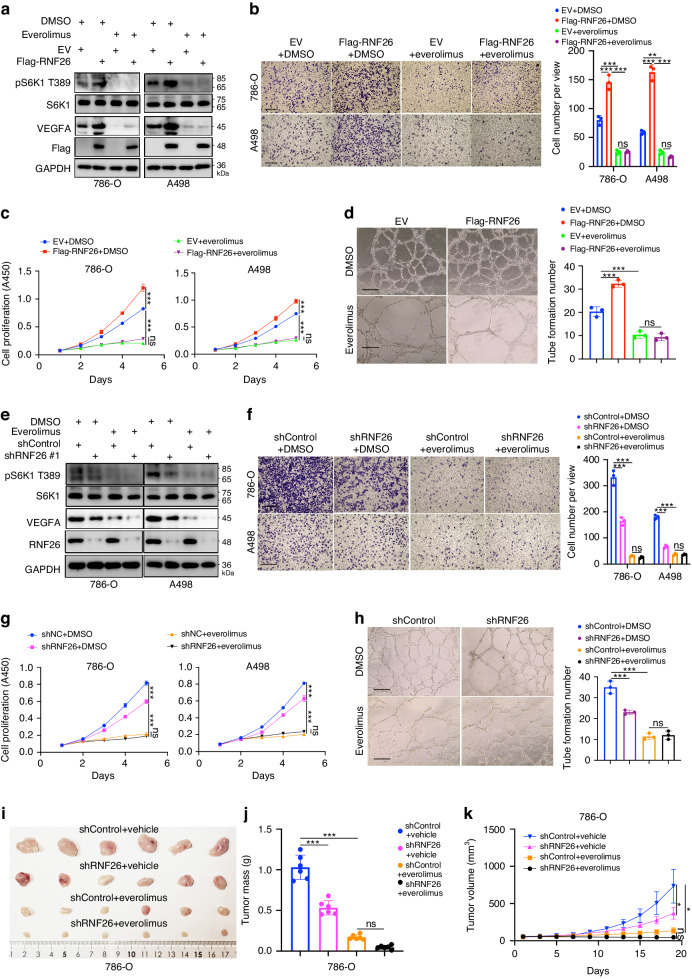
mTOR signaling is critical for determining the role of RNF26 in promoting ccRCC tumorigenesis. **a–d** 786-O and A498 cells were transfected with indicated plasmids for 24 h. Then, these cells were treated with or without Everolimus and subjected to western blotting, transwell, CCK-8, and tube formation assay. Data are presented as the mean ± SEM of three replicates. Ns, not significant; **, P < 0.01; ***, P < 0.001. **e–k** 786-O and A498 cells were infected with indicated shRNAs for 72 h. Then, these cells were treated with or without Everolimus and subjected to western blotting, transwell, CCK-8, tube formation, and nude mouse xenografts assay. Data are presented as the mean ± SEM of three replicates for in vitro assay and six replicates for in vivo assay. Ns, not significant; *, P < 0.05; ***, P < 0.001.

Since the mTOR signaling pathway is reported to regulate VEGFA expression and promote ccRCC angiogenesis and progression, we tested whether the mTOR signaling pathway is one of the key pathways mediating the cancer-related function of RNF26. First, we showed that everolimus treatment abolished the effect of RNF26 on modulating the expression of VEGFA and promoting the migration, proliferation, and angiogenesis of ccRCC cells (Fig. 5a–d). In contrast, everolimus treatment also diminished the changes in VEGFA induced by RNF26 knockdown in both 786-O and A498 cells (Fig. 5e). Then, we revealed that inactivation of the mTOR signaling pathway by everolimus not only attenuated the cell migration, proliferation and angiogenesis mediated by depletion of RNF26 in ccRCC cells (Figs. 5f–h) but also abolished the ability of RNF26 to enhance tumor growth in vivo (Figs. 5i–k). These data suggested that RNF26 promotes tumorigenesis mainly through the mTOR signaling pathway in ccRCC.

### RNF26 functions as an E3 ligase of TSC1 in ccRCC

**Fig. 6:**
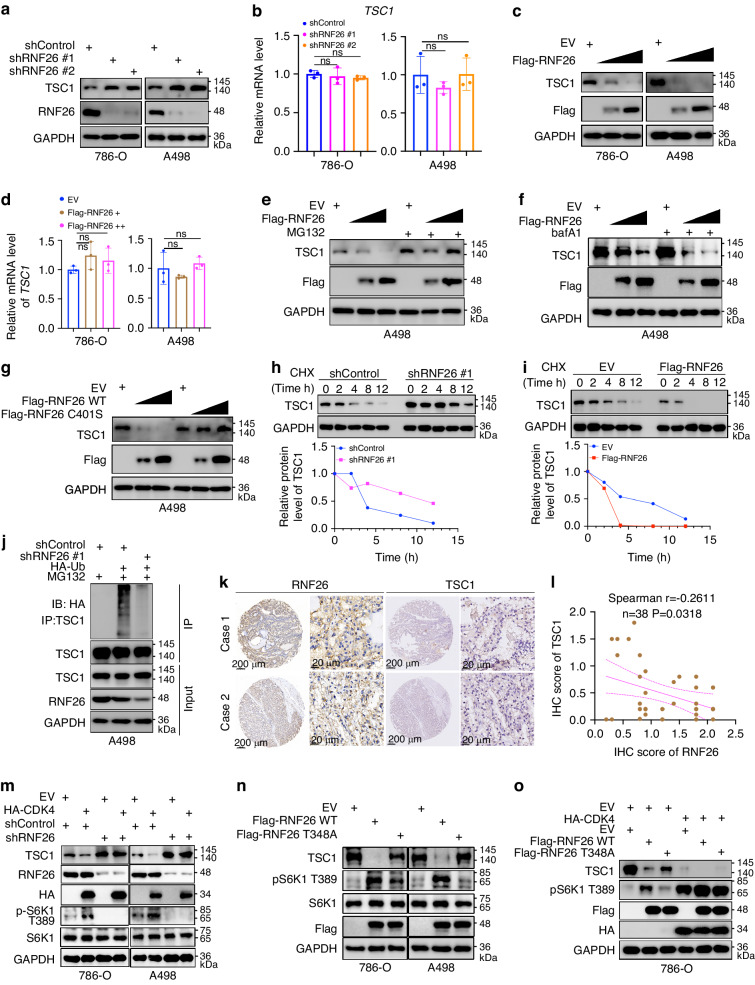
RNF26 functions as an E3 ligase of TSC1 in ccRCC. **a**, **b** 786-O and A498 cells were infected with indicated shRNAs for 72 h. Cells were harvested for western blotting (**a**) and RT-qPCR assay (**b**). Data are presented as the mean ± SEM of three replicates. Ns, not significant. **c**, **d** 786-O and A498 cells were transfected with indicated plasmids for 48 h. Cells were harvested for western blotting (**c**) and RT-qPCR assay (**d**). Data are presented as the mean ± SEM of three replicates. Ns, not significant. **e**, **f** A498 cells were transfected with indicated plasmids for 48 h. These cells were treated with or without MG132 or bafA1 and subjected to western blotting analysis. **g** A498 cells were transfected with indicated plasmids for 48 h and subjected to western blotting analysis. **h**, **i** 786-O cells were infected or transfected with the indicated shRNAs or plasmids for 72 h. Cells were treated with CHX, and cells were collected for western blotting analysis at different time points. **j** 786-O cells were infected with the indicated shRNAs for 72 h. Cells were harvested for immunoprecipitation and western blotting analysis. **k**, **l** IHC staining of the tissue microarray of ccRCC (n = 38) with RNF26 or TSC1 antibodies. **m–o** 786-O and A498 cells were transfected with indicated constructs for 72 h. Cells were harvested for western blotting analysis.

The above data indicate that RNF26 interacts with TSC1 (Fig. 2b) and that RNF26 overexpression seems to decrease the expression of TSC1 (Fig. 2c). Since TSC1 is a negative regulator of the mTOR signaling pathway [28], we wondered whether RNF26 promotes the degradation of TSC1 in ccRCC. We demonstrated that knockdown of RNF26 increased the protein level but not the mRNA level of TSC1 in 786-O and A498 cells (Fig. 6a, b). Overexpression of RNF26 decreased the protein level but not the mRNA level of TSC1 in ccRCC cells (Fig. 6c, d), and this effect could be attenuated by a proteasome inhibitor (MG132) but not a lysosomal inhibitor (BafA1) (Fig. 6e, f). Moreover, we demonstrated that the RNF26 catalytically dead mutant (RNF26 C401S) could not decrease the expression of TSC1 in A498 cells (Fig. 6g). In addition, knockdown of RNF26 prolonged the half-life of TSC1 and decreased the polyubiquitination of TSC1, but overexpression of RNF26 shortened the half-life of TSC1 in A498 cells (Fig. 6h–j). Furthermore, the results of the IHC staining assay of the ccRCC tissue microarray revealed that RNF26 expression was negatively correlated with TSC1 expression (Spearman r = -0.2611, P = 0.0318, n = 38) (Fig. 6k, l).

Given that CDK4/6 inhibitors regulate the stability of TSC1 (Fig. 1) and that CDK4-mediated phosphorylation of RNF26 promotes RNF26 binding with TSC1 in ccRCC cells (Fig. 2), we showed that knockdown of RNF26 diminished the TSC1 protein level change induced by overexpression/knockdown of CDK4 or treatment with CDK4/6 inhibitors in 786-O and A498 cells (Fig. 6m, Supplementary Fig. 3a, b). Overexpression of the RNF26 T348A mutant had less of an effect on the protein levels of TSC1 and p-S6K1 (T389) than overexpression of wild-type RNF26 with or without overexpression of CDK4 in ccRCC cells (Fig. 6n, o). In contrast, overexpression of the RNF26 T348D mutant had a more pronounced impact on the protein levels of TSC1 and p-S6K1 (T389) compared to the overexpression of wild-type RNF26 on the protein levels of TSC1 and p-S6K1 (T389) (Supplementary Fig. 3c). Moreover, in 786-O and A498 cells, overexpression of RNF26 T348D had a more pronounced impact on the proliferation and angiogenesis of ccRCC cells compared to overexpression of RNF26 T348A (Supplementary Fig. 4a–c). These data suggest that RNF26 functions as an E3 ligase of TSC1 and is involved in regulating the stability of TSC1 induced by CDK4.

### The RNF26/TSC1 axis regulates the progression and angiogenesis of ccRCC

Next, we examined whether RNF26 regulates tumorigenesis via TSC1 in ccRCC. First, we showed that knockdown of TSC1 diminished the changes in VEGFA and p-S6K1 (T389) induced by overexpression of RNF26 in 786-O and A498 cells (Supplementary Fig. 5a). Transwell, CCK-8 and tube formation assays demonstrated that depletion of TSC1 attenuated the effect of RNF26 overexpression on increasing tumor cell migration, proliferation, and angiogenesis (Supplementary Fig. 5b–d). On the other hand, we also revealed that compared to RNF26 knockdown alone, coknockdown of TSC1 and RNF26 did not further decrease VEGFA and p-S6K1 (T389) levels in 786-O and A498 cells (Supplementary Fig. 5e). Similarly, knockdown of TSC1 attenuated the changes in cell migration, proliferation and angiogenesis mediated by knockdown of RNF26 in ccRCC cells (Supplementary Fig. 5f–h). In addition, a nude mouse model demonstrated that the tumor-promoting effect of RNF26 could be diminished by knockdown of TSC1 (Supplementary Fig. 5i–k). Thus, our data suggest that RNF26 promotes the progression and angiogenesis of ccRCC, in part through TSC1.

### The RNF26/TSC1 axis modulates the sensitivity of ccRCC to CDK4/6 inhibitors and mTOR inhibitors

The combination of a CDK4/6 inhibitor and an mTOR inhibitor is a promising strategy for the treatment of ccRCC [18]. mTOR inhibitors are considered antiangiogenic drugs for ccRCC treatment [29, 30]. Since the RNF26/TSC1 axis connects the CDK4 and mTOR signaling pathways, we wondered whether the RNF26/TSC1 axis regulates sensitivity to CDK4/6 inhibitors and mTOR inhibitors. First, we found that knockdown of TSC1 diminished the changes in VEGFA expression and angiogenesis induced by treatment with palbociclib (Supplementary Fig. 6a, b). We demonstrated that palbociclib treatment decreased the IC50 of everolimus, and this effect was attenuated after knockdown of TSC1 in 786-O and A498 cells (Supplementary Fig. 6c). Subsequent colony formation assays also showed that repression of TSC1 decreased the anticancer effect of the combination of palbociclib and everolimus (Supplementary Fig. 6d). Moreover, we noticed a similar effect on changes in VEGFA and p-S6K1 (T389) levels: knockdown of RNF26 in ccRCC cells altered the sensitivity to palbociclib and everolimus (Supplementary Fig. 6e–h). Taken together, these results indicated that the CDK4/RNF26/TSC1 axis regulates the anticancer efficacy of CDK4/6 inhibitors and mTOR inhibitors in ccRCC. CDK4/6 inhibitors regulate the mTOR signaling pathway through the CDK4/RNF26/TSC1 axis in ccRCC (Supplementary Fig. 7).

## Discussion

CDK4 and CDK6 are related kinases that play important regulatory roles in the G1/S transition phase of the cell cycle. CDK4/6 form a complex with their ligand protein cyclin D, facilitating the progression of the cell cycle. By regulating key mechanisms such as the cell cycle, transcription factors, and cell apoptosis, CDK4/6 participates in the regulation of tumor occurrence and development. Research on CDK4/6 contributes to providing a theoretical basis for antitumor treatment strategies [19]. Currently, the CDK4/6-retinoblastoma (RB) pathway is widely recognized as an important pathway involved in tumor regulation. However, in certain types of tumors, the activation of CDK4/6 may not only depend on the RB protein but may also be regulated by other important factors. For example, the interaction between CDK4/6 and the transcription factor MYC plays a synergistic role in promoting tumor cell proliferation and metastasis [31]. CDK4/6 also phosphorylates and activates the transcription factor FOXM1, further promoting cell proliferation and tumor development [32]. TSC1 is a key gene in the tuberous sclerosis complex (TSC). Under normal conditions, the TSC1-TSC2 complex inhibits the activity of Rheb, thereby suppressing the mTOR signaling pathway. When the TSC1 gene is mutated or its function is impaired, it may lead to excessive activation of the mTOR signaling pathway, promoting cancer cell proliferation, metastasis, and tumor development [33]. However, the potential role of TSC1 in the progression of ccRCC tumors remains unclear. In this study, we found that CDK4/6 inhibitors are involved in regulating the protein stability of TSC1 in ccRCC.

RNF26 is a member of the RING finger protein family and is located in the human chromosome 11q23 region. It is characterized by the presence of a C-terminal RING finger domain [34]. According to reports, RNF26 limits the response to type I interferon by promoting the autophagic degradation of IRF3. RNF26 also stabilizes TMEM173/STING, which catalyzes the formation of K11-linked polyubiquitin chains at lysine 150 to prevent its degradation by RNF5 [35]. RNF26 is also an E3 ubiquitin ligase located on the endoplasmic reticulum within the cell. By mediating the ubiquitination of SQSTM1, it attracts various vesicle adapter ubiquitin-binding domains, ultimately regulating the efficiency of intranuclear trafficking [36]. Previous studies have shown that RNF26 is upregulated in HL-60 (acute promyelocytic leukemia), HeLa S3 (cervical cancer), SW480 (colorectal cancer), and MKN7 (gastric cancer) cells [34]. In urinary system tumors, recent reports have revealed the RNF26/CBX7 axis, where RNF26 promotes the degradation of CBX7, regulating the TNF signaling pathway in ccRCC and promoting ccRCC tumor growth [26]. Additionally, it has been reported that abnormal overexpression of FOXM1 leads to upregulation of RNF26 in bladder cancer cells through the MuvB complex. RNF26 can interact with p57, reducing its stability and enhancing the invasiveness of bladder cancer cells. The FOXM1/RNF26/p57 axis has been proposed as a new therapeutic target for bladder cancer [25]. Interestingly, we identified RNF26 as a regulator of TSC1 protein expression. By constructing an RNF26-T348A mutant, we discovered that CDK4 phosphorylates RNF26 at the T348 site, enhancing the interaction between TSC1 and RNF26. In nude mouse studies and cell experiments, we observed that RNF26 contributes to the progression and angiogenesis of ccRCC. Furthermore, we demonstrated that RNF26 plays a critical role in activating the mTOR signaling pathway in ccRCC. Due to the reported regulation of VEGFA expression and promotion of angiogenesis and progression in ccRCC by the mTOR signaling pathway, we conducted experiments to verify whether the mTOR pathway is a crucial mediator of the functions of RNF26 in ccRCC. We found that inhibiting the mTOR signaling pathway can eliminate the regulatory effects of RNF26 on VEGFA expression, ccRCC cell migration, proliferation, and angiogenesis. Moreover, RNF26, an E3 ligase of TSC1, facilitates the degradation of TSC1 in ccRCC and, to a certain extent, promotes the progression and angiogenesis of ccRCC through TSC1.

CDK4/6 inhibitors have been widely studied internationally, and there are relevant studies on their application in ccRCC. Wogonin or palbociclib, when applied to 786-O cells resistant to sunitinib, can effectively reverse RCC resistance to sunitinib [37]. Both abemaciclib alone and in combination with sunitinib can reduce cell viability and promote apoptosis in ccRCC cells. In addition, abemaciclib alone or in combination with sunitinib led to the regression of 786-O xenografts, and tumor regression was also observed when abemaciclib was added after sunitinib pretreatment [38]. Currently, a phase I clinical trial (NCT03905889) is underway to investigate combination therapy with abemaciclib and sunitinib for the treatment of metastatic RCC (US National Library of Medicine. ClinicalTrials.gov https://ClinicalTrials.gov/show/NCT03905889 (2022)). There is significant exploratory value in the combination of multiple drugs for the treatment of tumors. It has been reported that the combined use of CDK4/6 inhibitors and mTOR inhibitors can provide greater benefits to patients in the treatment of breast cancer [39]. Therefore, we explored the interrelationship between CDK4/6 inhibitors and mTOR inhibitors in ccRCC and discovered that the RNF26/TSC1 axis plays a bridging role between the CDK4 and mTOR signaling pathways. CDK4/6 inhibitors can regulate the mTOR signaling pathway in ccRCC through the CDK4/RNF26/TSC1 axis. The CDK4/RNF26/TSC1 axis regulates the anticancer effects of CDK4/6 inhibitors and mTOR inhibitors in ccRCC.

Although CDK4/6 inhibitors have shown potential clinical value in the treatment of RCC, further research is still needed to determine their optimal use, including drug dosage, combination therapy, and treatment duration. In addition, the safety and tolerability of these inhibitors need to be further evaluated, as known adverse events include bone marrow suppression, gastrointestinal adverse reactions, abnormal liver function, and adverse reactions in the skin and subcutaneous tissues [40]. There are several limitations to this study. On the one hand, we did not combine CDK4/6 inhibitors or other drugs to further explore their therapeutic effects in animal models of renal cancer; we used the A498 and 786-O cell lines in this study, and we may have to validate the findings in more renal cancer cell lines in the future.

Collectively, our findings indicate that CDK4/6 inhibitors can increase the level of the TSC1 protein in renal cancer cells and regulate its stability. Moreover, overexpression of CDK4 can decrease the level of the TSC1 protein. Additionally, RNF26 interacts with TSC1 and regulates the proliferation, migration, and angiogenesis of renal cancer cells through the mTOR signaling pathway. RNF26 serves as an E3 ligase for TSC1 in ccRCC, participating in regulating TSC1 stability. This finding suggested that RNF26 promotes the progression and angiogenesis of ccRCC to some extent by negatively regulating TSC1. Therefore, our findings reveal a new CDK4/RNF26/TSC1 axis that regulates the anticancer efficacy of CDK4/6 inhibitors and mTOR inhibitors in ccRCC.

### Supplementary information

Supplementary Information

## Data Availability

The datasets used and/or analyzed during the current study are available from the corresponding authors on reasonable request. The data generated in this study are publicly available in Gene Expression Omnibus (GEO) at GSE23816625.
