# Supplementary material for: CDK4/6 inhibitors dephosphorylate RNF26 to stabilize TSC1 and increase the sensitivity of ccRCC to mTOR inhibitors
Source: Br J Cancer. 2024 Jun 18;131(3):444–56. doi: 10.1038/s41416-024-02750-3 (PMC11300639; doi:10.1038/s41416-024-02750-3)
Supplement: Supplementary file 1 — Supplementary Information [file 41416_2024_2750_MOESM1_ESM.docx]

**CDK4/6 inhibitors dephosphorylate RNF26 to stabilize TSC1 and increase the sensitivity of ccRCC to mTOR inhibitors**

Xinlin Liu, Wei Li, Lu Yi, Jianxi Wang, Wentao Liu, Hongtao Cheng, Shangqing Ren


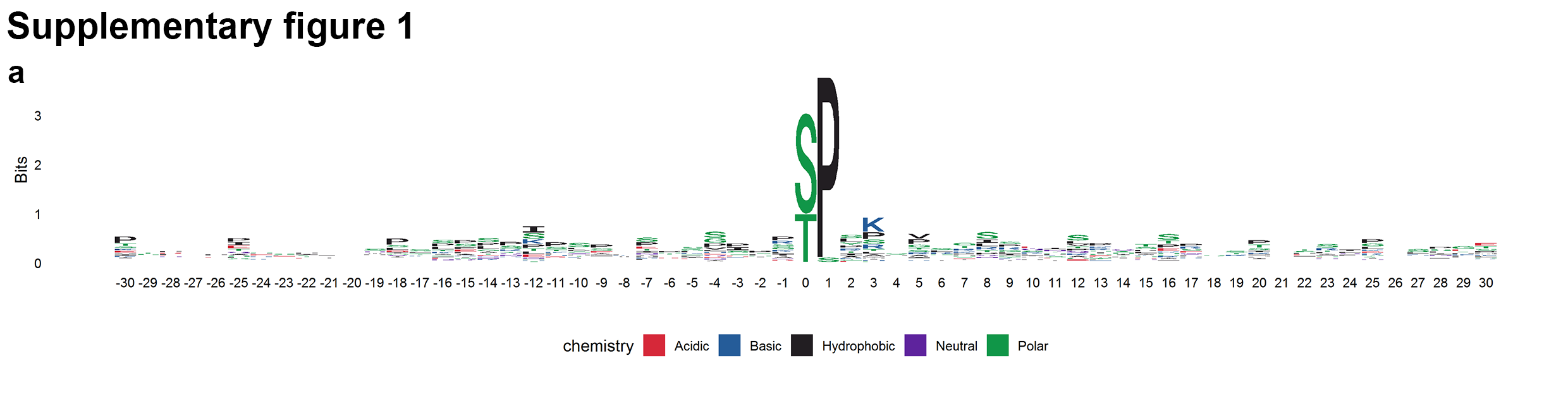


**Supplementary figure 1**

**a,** analysis of a site (Tyrosine, T) among the amino acid sequence of RNF26 could be phosphorylated by CDK.


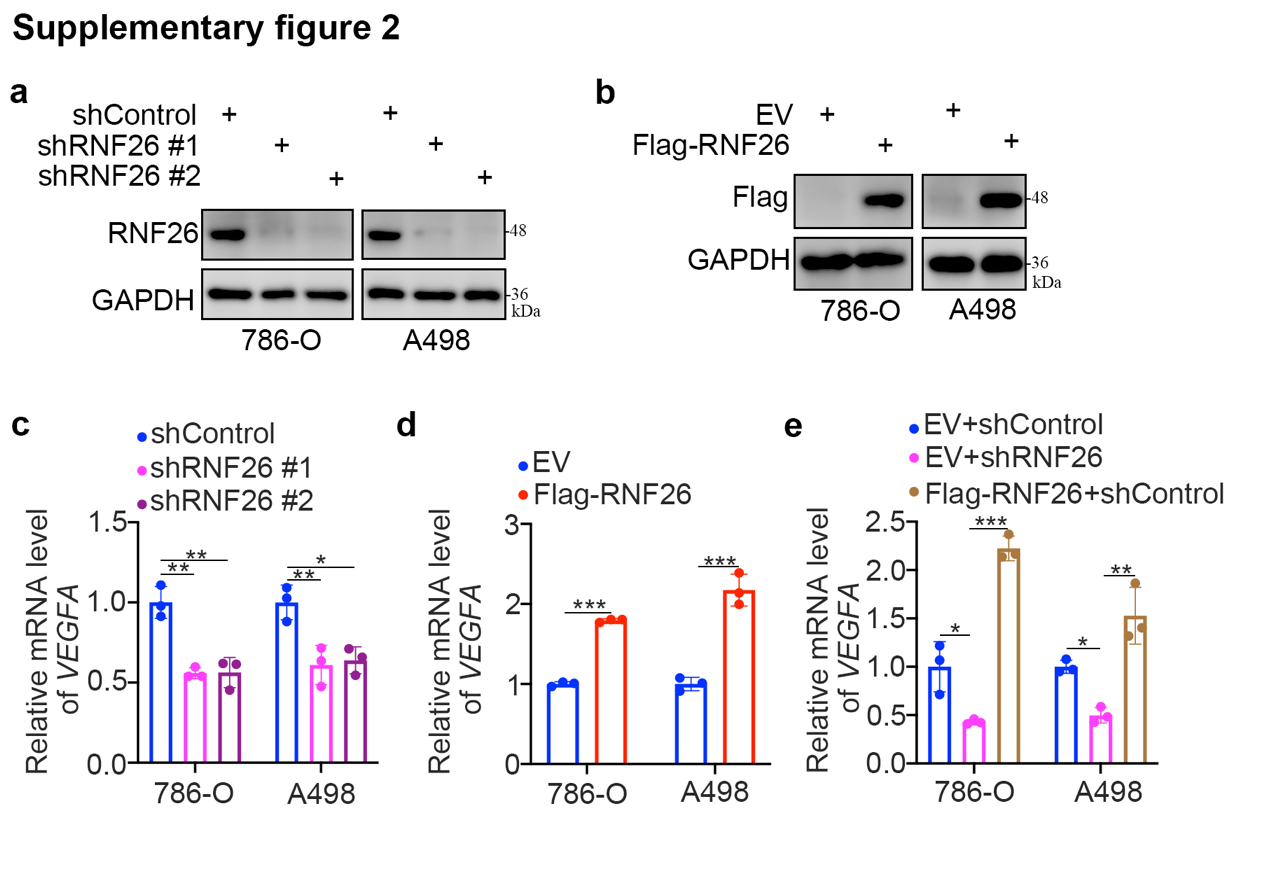


**Supplementary figure 2.**

**a and b,** 786-O and A498 cells were infected or transfected with indicated shRNAs or plasmids for 72 h or 48 h. Cells were harvested for western blotting analysis. **c-e,** 786-O and A498 cells were transfected with indicated constructs for 72 h. Cells were harvested for RT-qPCR assay. Data are presented as the mean ± SEM of three replicates. *, P < 0.05; **, P < 0.01; ***, P < 0.001.


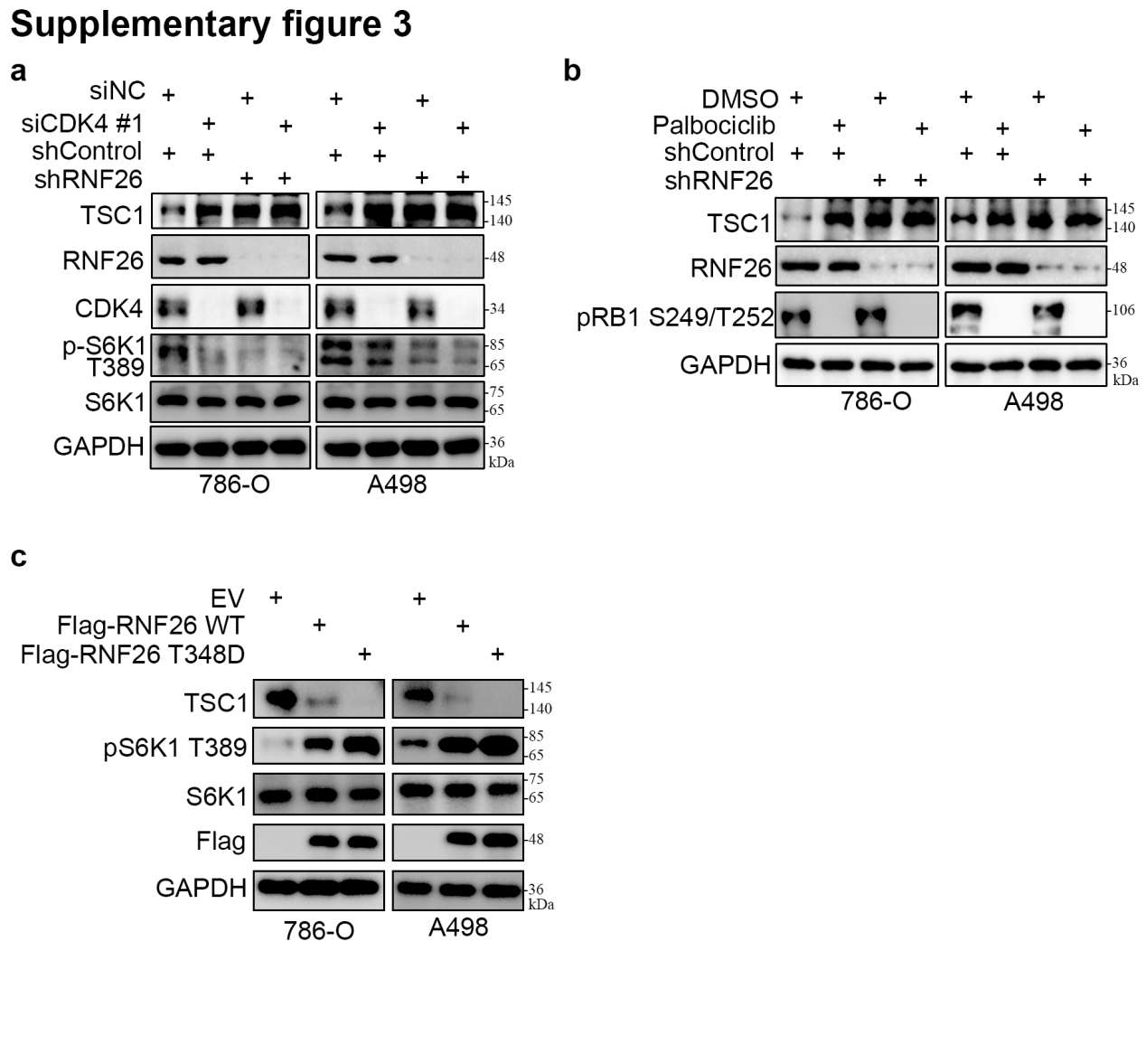


**Supplementary figure 3.**

**a-c,** 786-O and A498 cells were transfected with indicated constructs for 72 h. Cells were harvested for western blotting analysis.

_
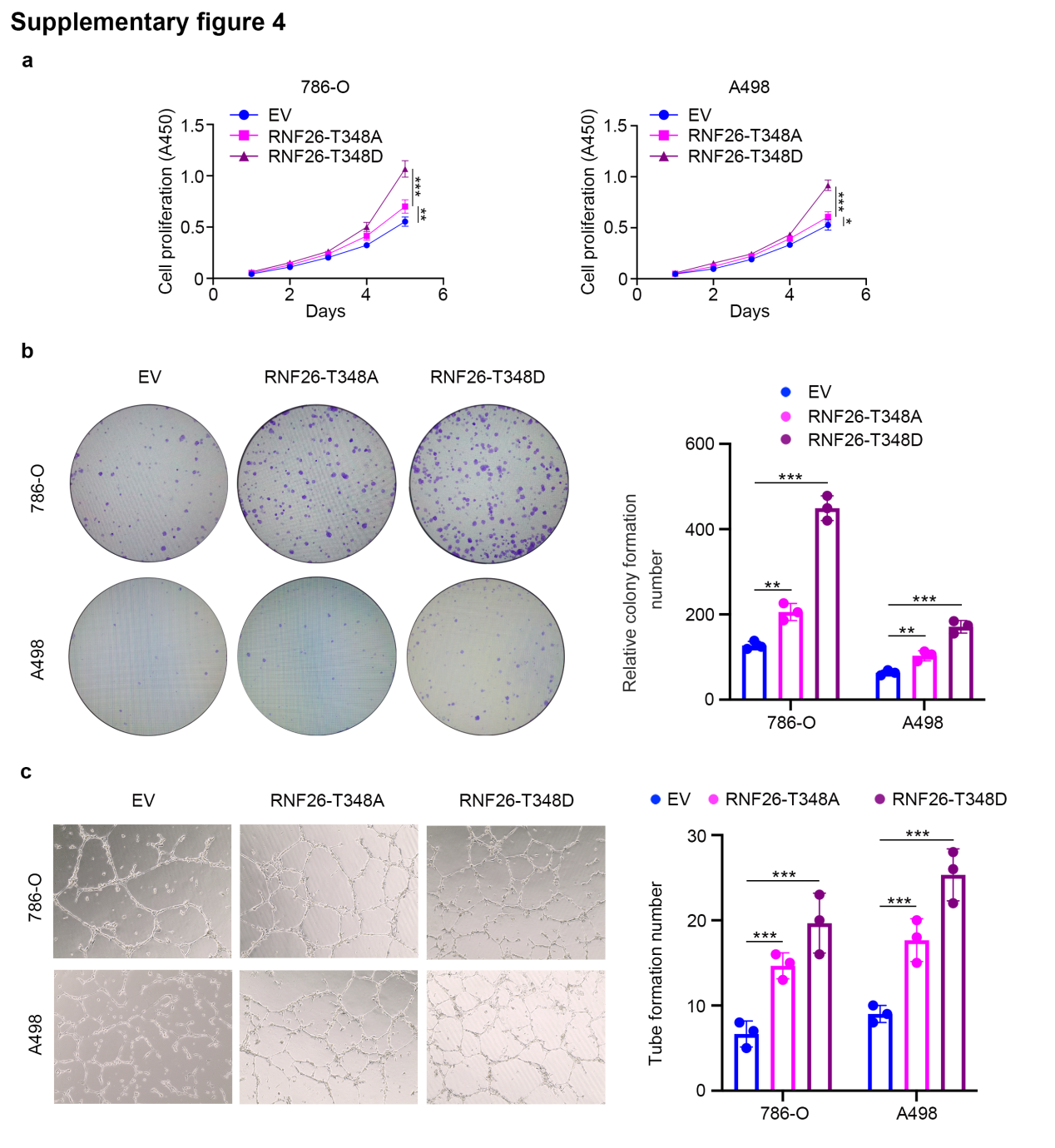
_

**Supplementary figure 4.**

**a-c,** 786-O and A498 cells were transfected with indicated constructs for 72 h. Then, these cells were subjected to CCK-8, colony formation assay and tube formation assay. Data are presented as the mean ± SEM of three replicates. *, P < 0.05; **, P < 0.01; ***, P < 0.001.


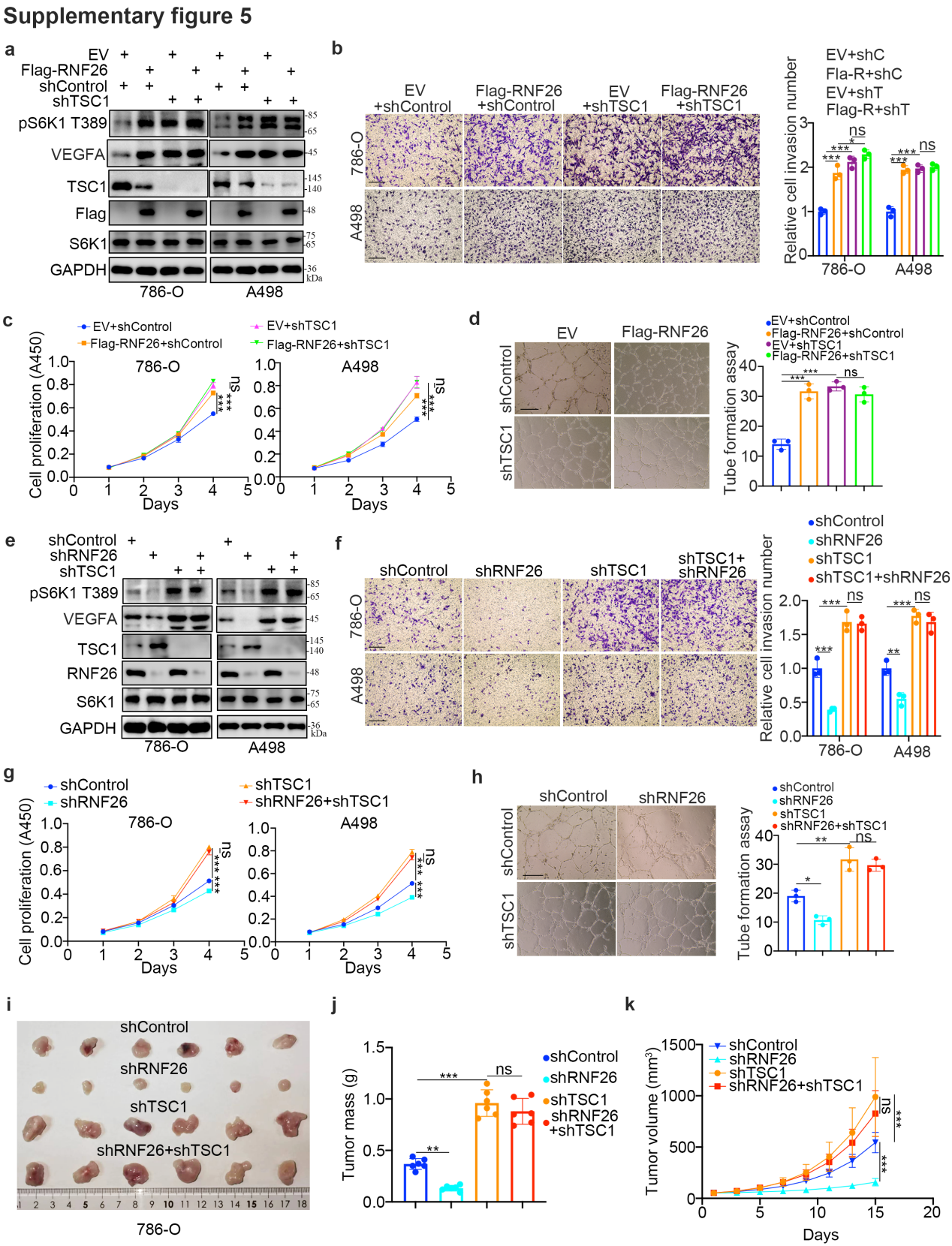


**Supplementary figure 5. The RNF26/TSC1 axis regulates the progression and angiogenesis of ccRCC**

**a-d,** 786-O and A498 cells were transfected with indicated constructs for 72 h. Then, these cells were subjected to western blotting, transwell, CCK-8, and tube formation assay. Data are presented as the mean ± SEM of three replicates. Ns, not significant; *, P < 0.05; ***, P < 0.001. **e-k,** 786-O and A498 cells were transfected with indicated constructs for 72 h. Then, these cells were subjected to western blotting, transwell, CCK-8, tube formation, and nude mouse xenografts assay. Data are presented as the mean ± SEM of three replicates. Ns, not significant; **, P < 0.01; ***, P < 0.001.


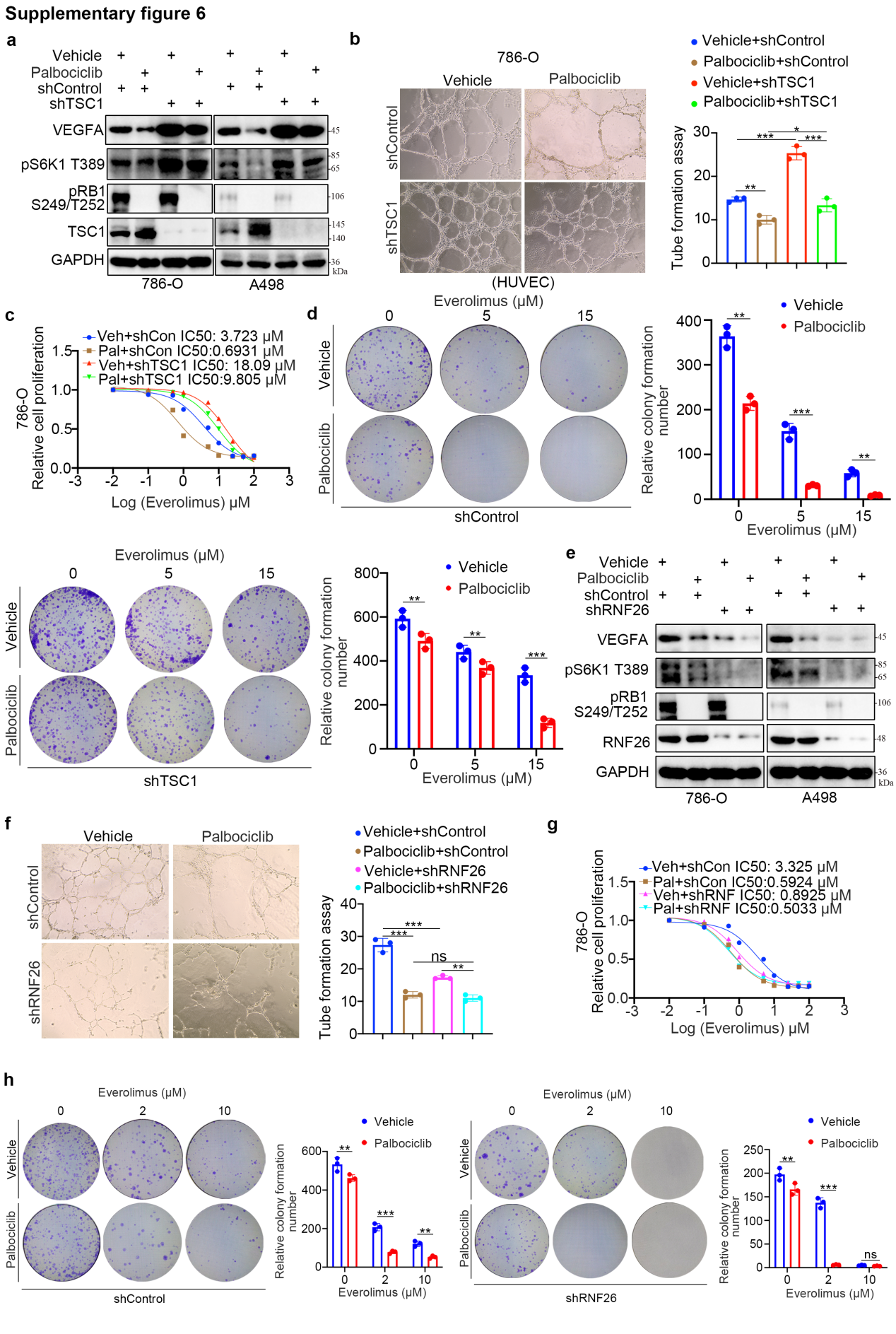


**Supplementary figure 6. The RNF26/TSC1 axis modulates the sensitivity of ccRCC to CDK4/6 inhibitors and mTOR inhibitors**

**a-d,** 786-O and A498 cells were transfected with indicated constructs for 72 h. Then, these cells were treated with or without Palbociclib (5 μM) and subjected to western blotting, tube formation, CCK-8, and colony formation assay. Data are presented as the mean ± SEM of three replicates. *, P < 0.05; **, P < 0.01; ***, P < 0.001. **e-h**, 786-O and A498 cells were transfected with indicated constructs for 72 h. Then, these cells were treated with or without Palbociclib (5 μM) and subjected to western blotting, tube formation, CCK-8, and colony formation assay. Data are presented as the mean ± SEM of three replicates. ns, not significant; **, P < 0.01; ***, P < 0.001.


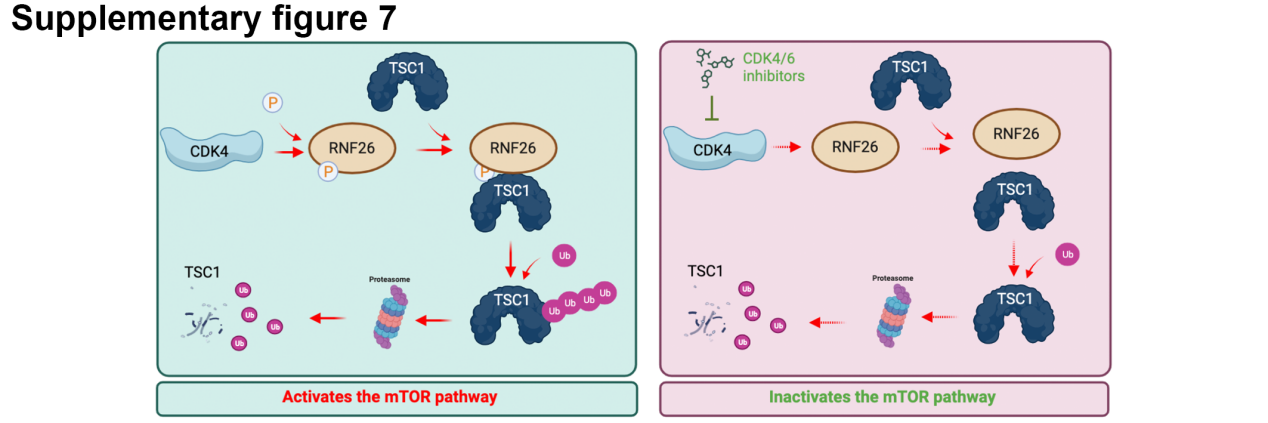


**Supplementary figure 7.**

A model depicting that CDK4 phosphorylates RNF26 to promote RNF26 binds with TSC1, destabilizes TSC1 and activates the mTOR signaling pathway, the CDK4/6 inhibitor impedes this process and enhanced the anti-cancer efficacy of the mTOR inhibitors in ccRCC.


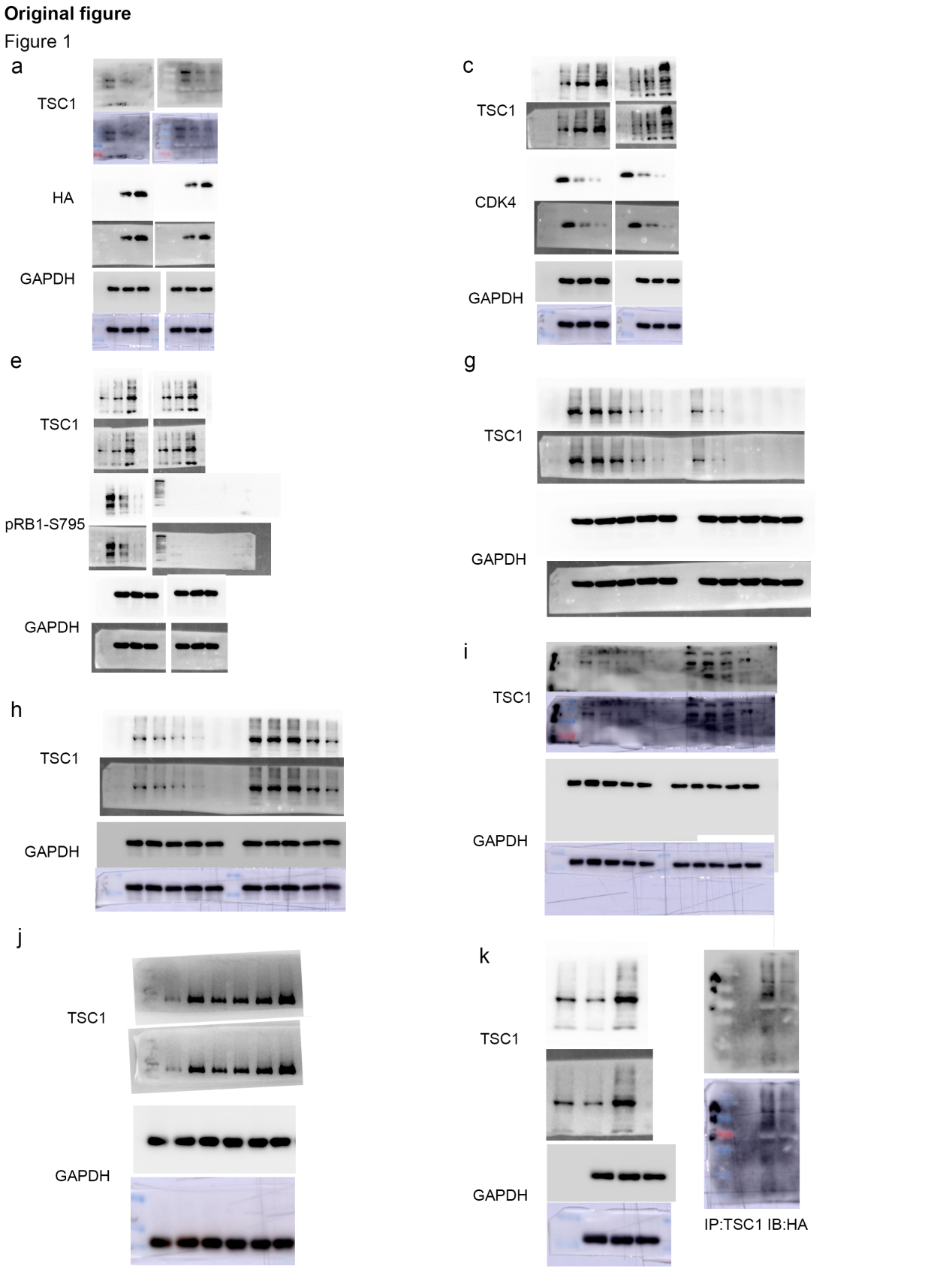


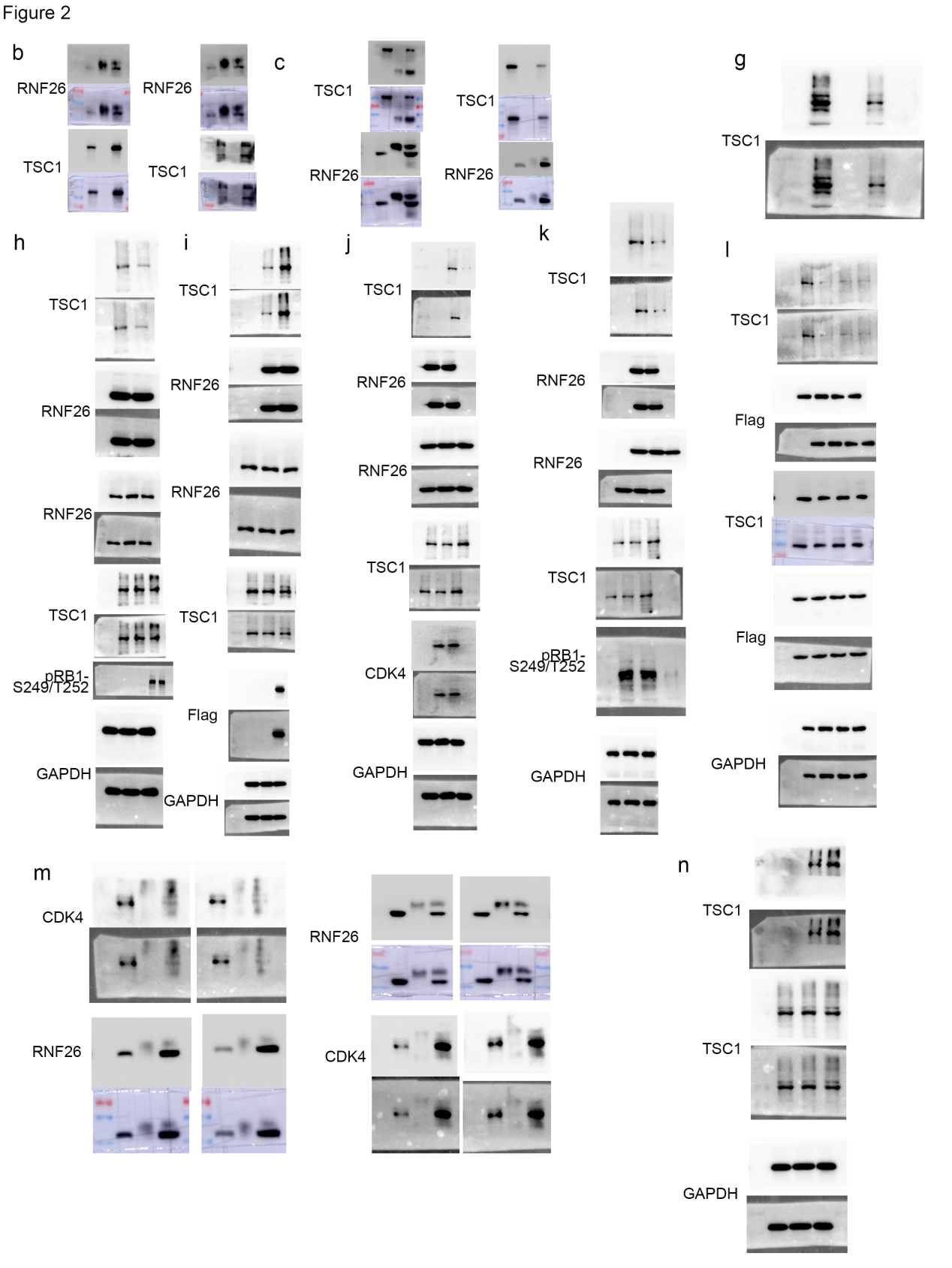


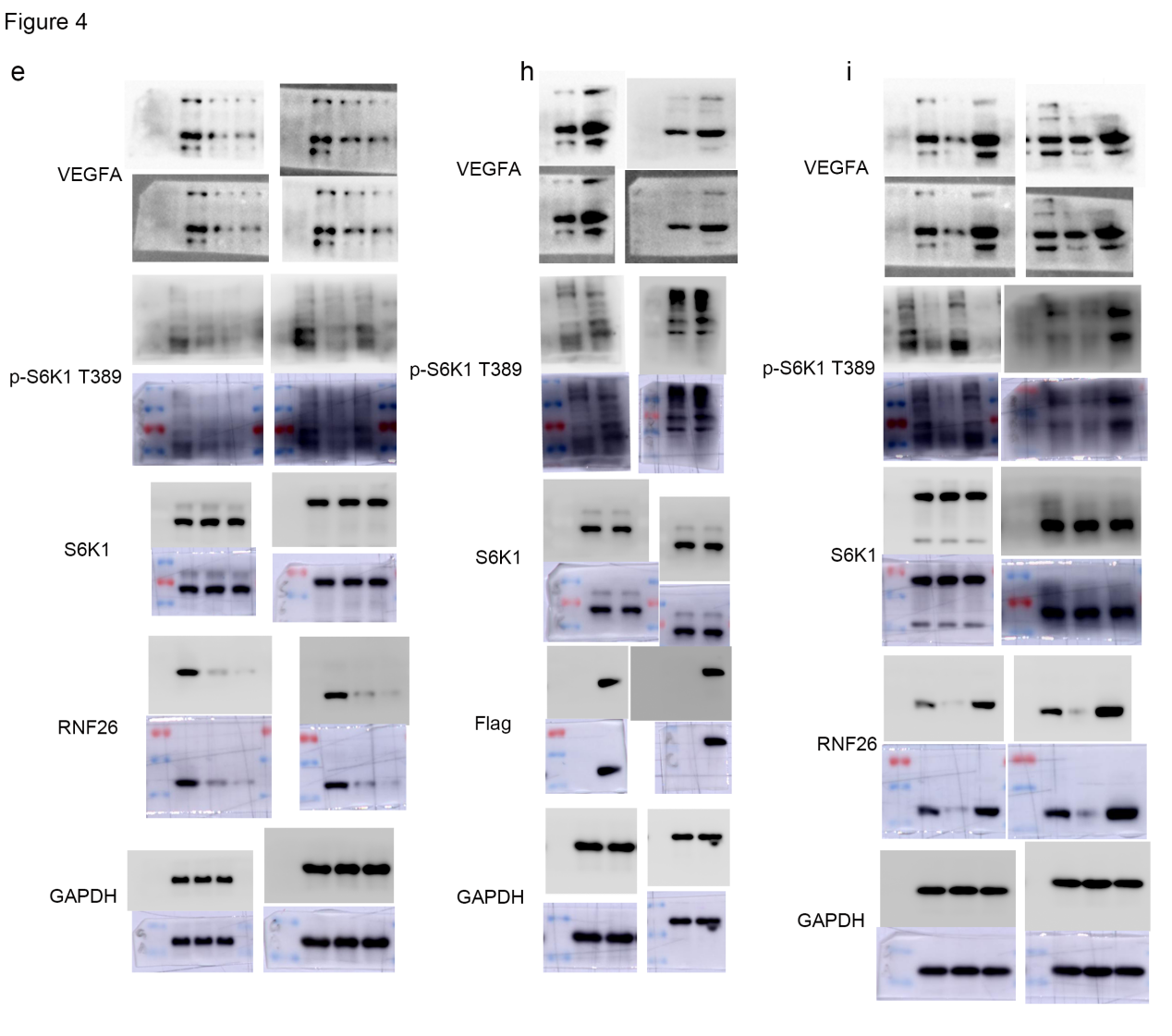


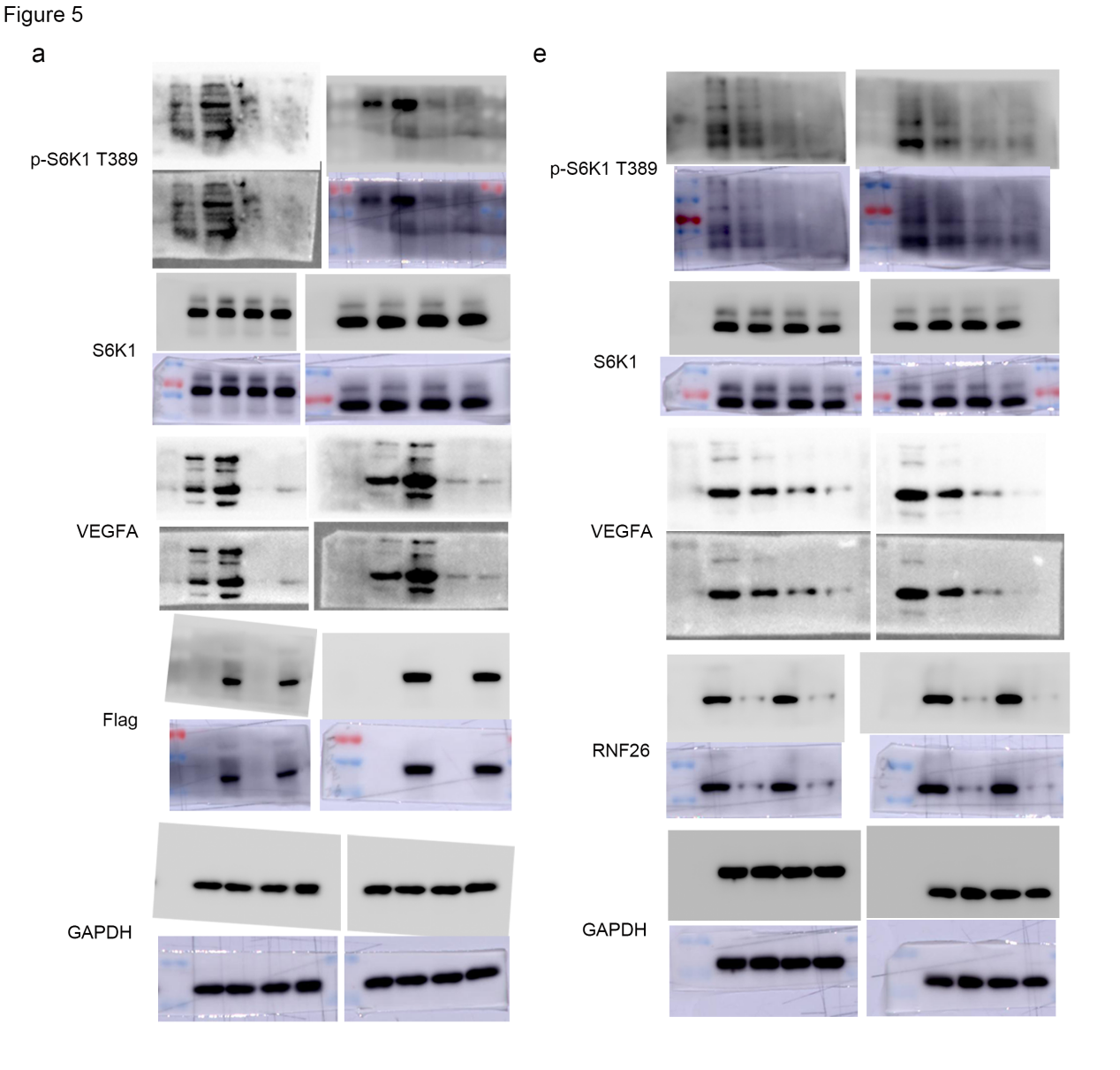


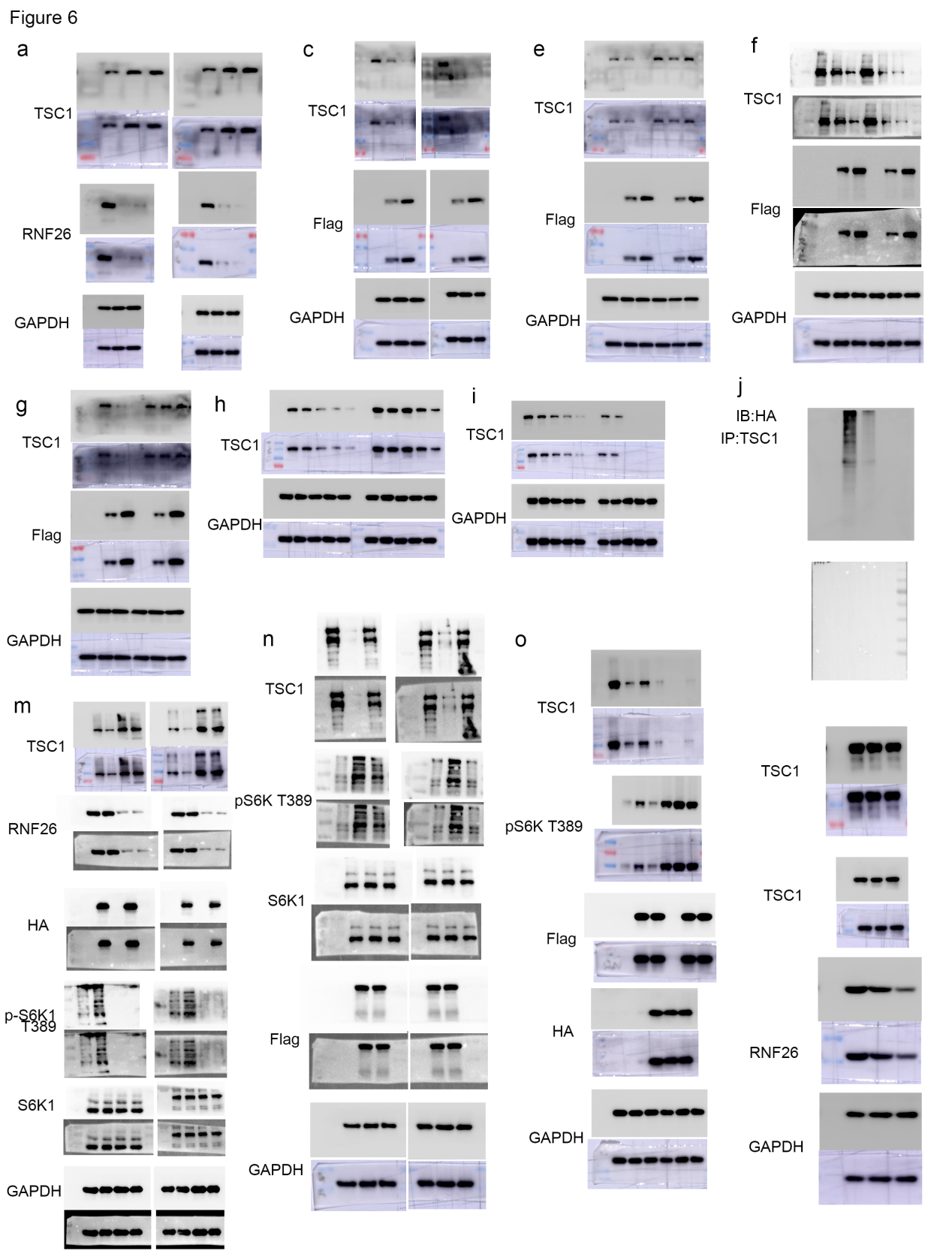


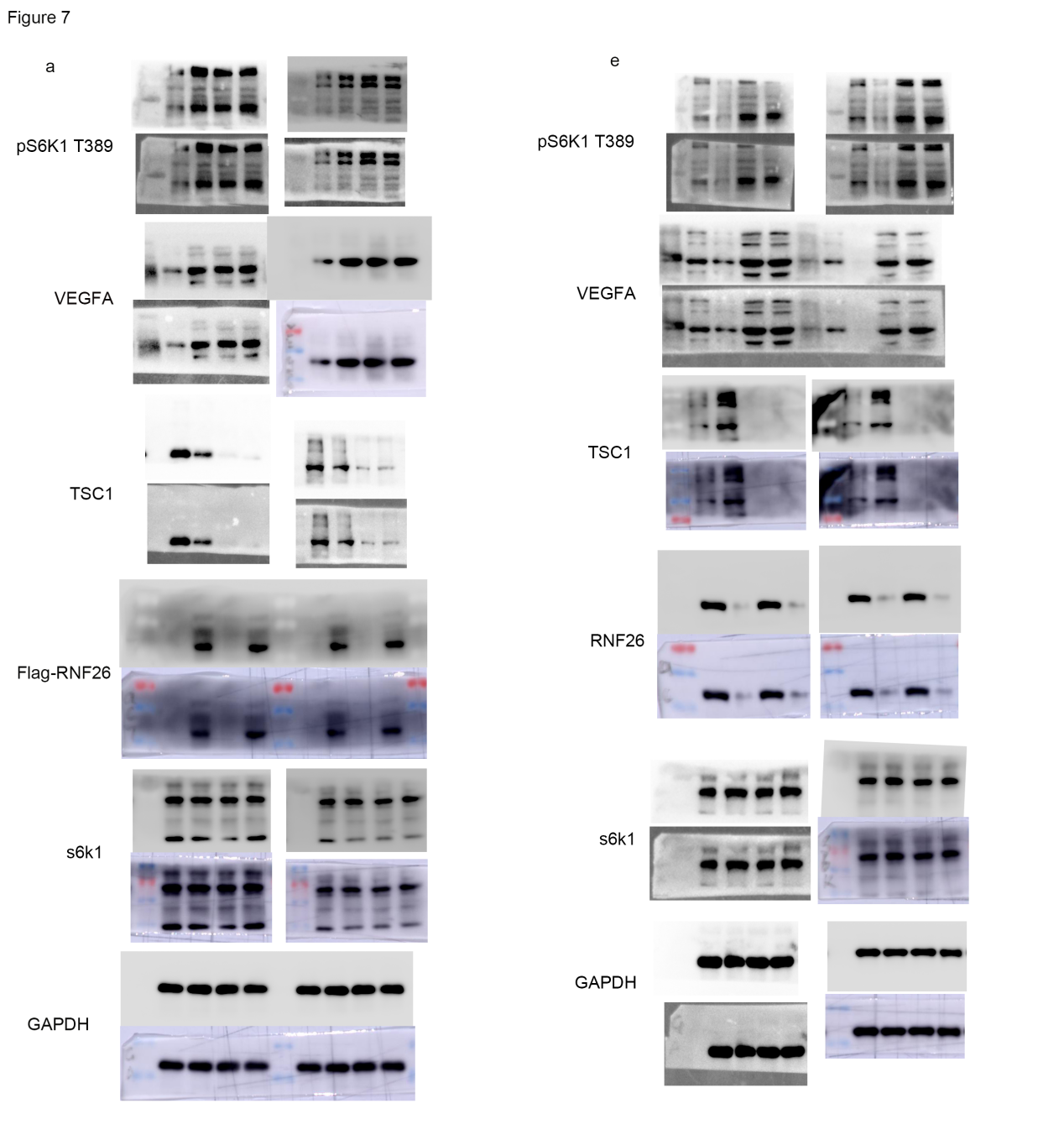


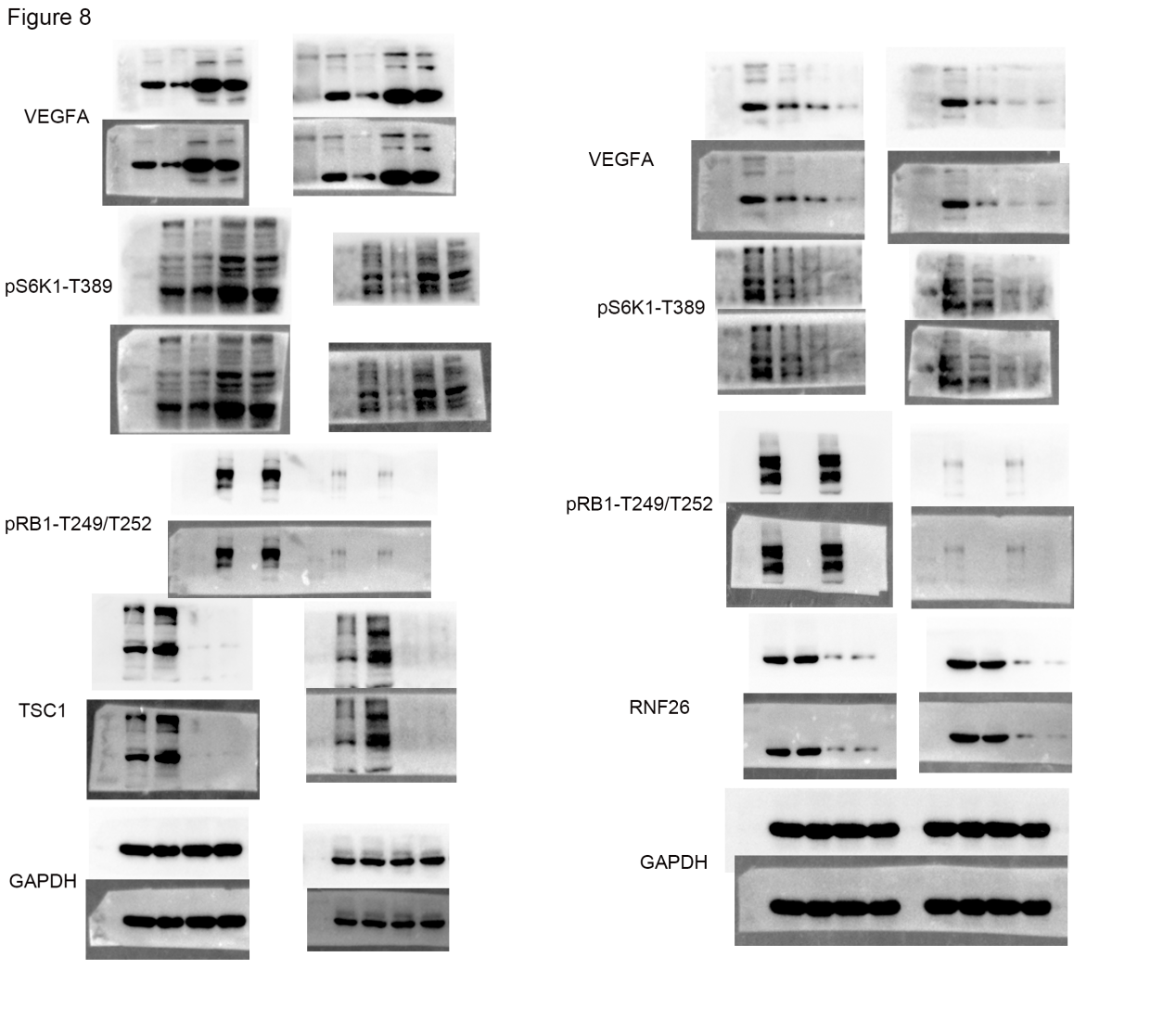


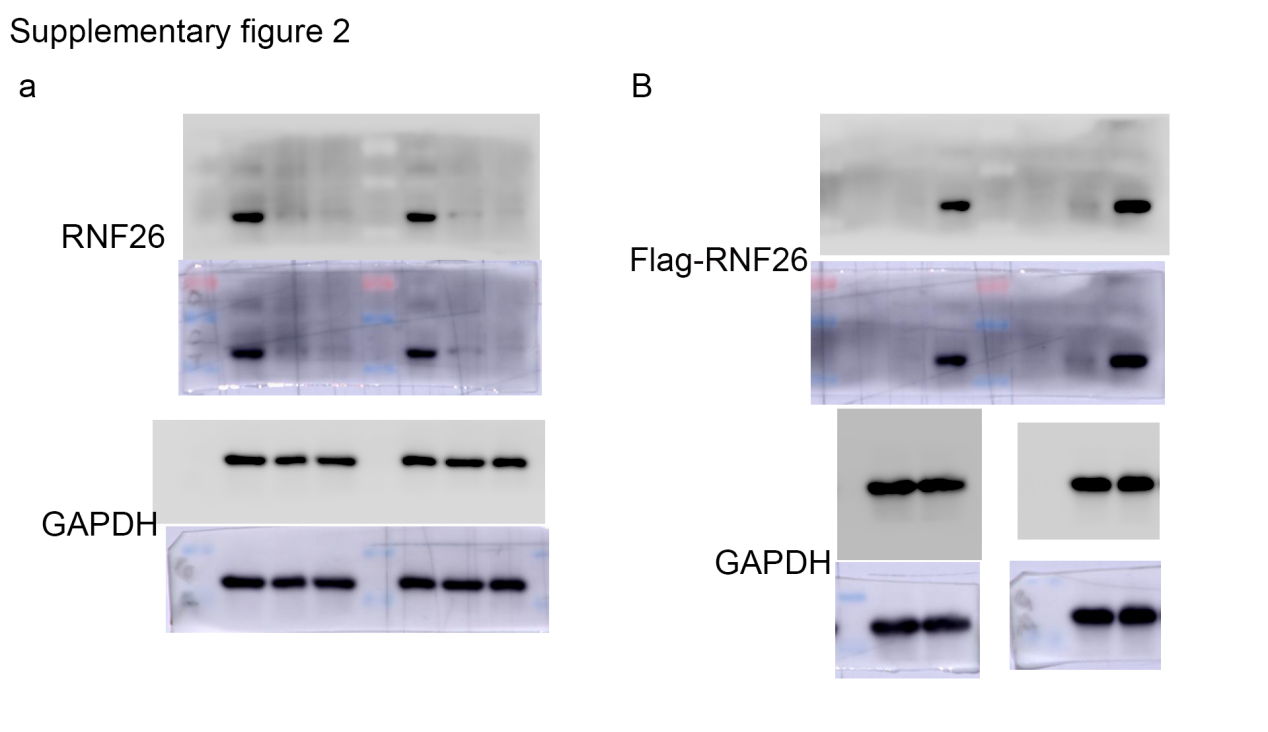


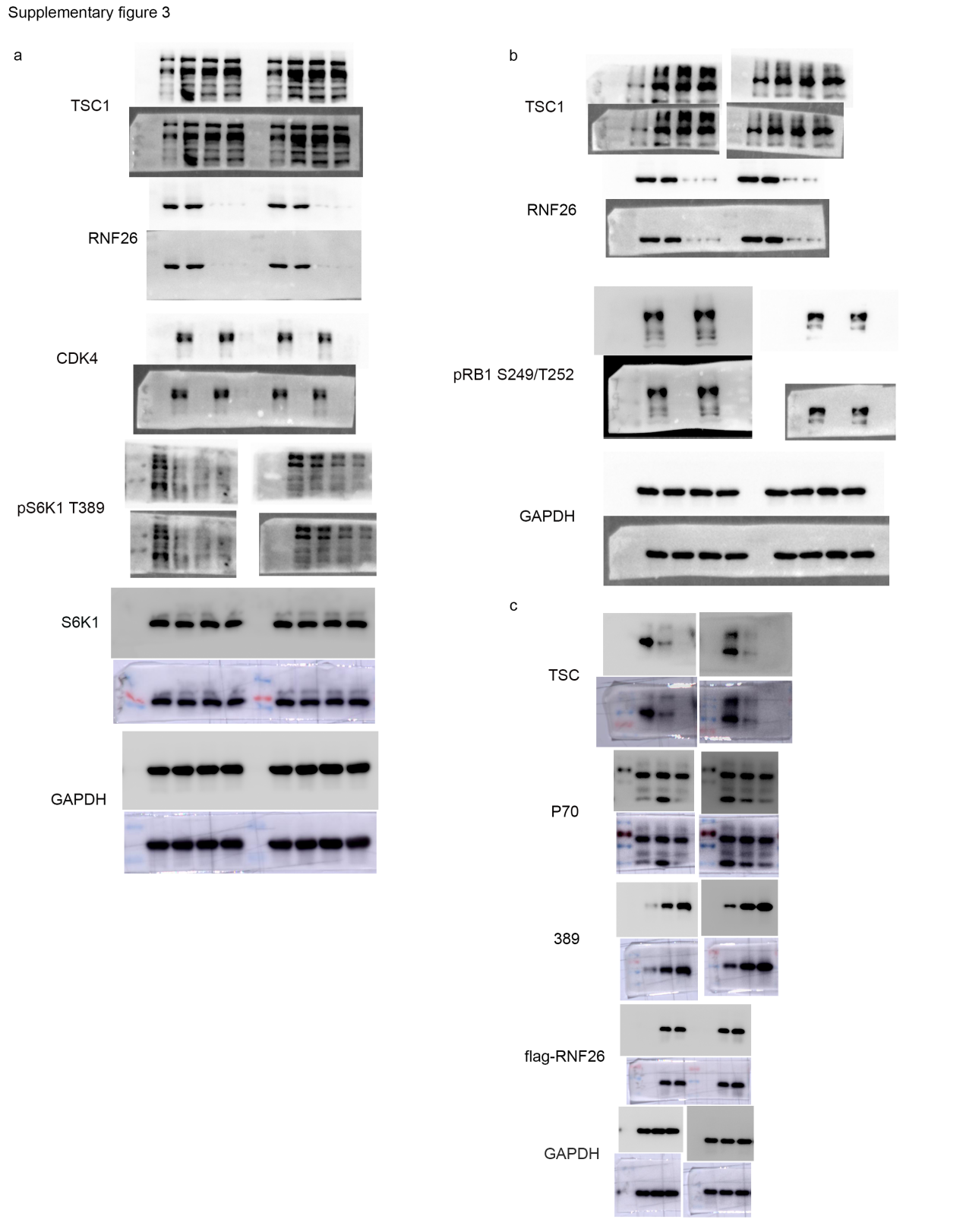


**Original figure.**

The original data of the western blot.

**Supplementary Material and Methods**

**Glutathione S-transferase pull-down assay**

Cells were lysed with 1 × RIPA lysis buffer (P0013B, Beyotime, shanghai, China) for 30 minutes at 4 ℃. Glutathione S-transferase (GST) fusion proteins were immobilized on BeyoMag™ Anti-GST Magnetic Beads (P2138, Beyotime, shanghai, China). After washed with 1 × RIPA lysis buffer, the beads were incubated with cell lysates for 4 hours. The beads were then washed four times with 1 × RIPA lysis buffer and resuspended in loading buffer. The bound proteins were subjected to SDS/PAGE and Western blotting.

**Liquid chromatography-tandem mass spectrometry/mass spectrometry analysis**

For the mass spectrometry analysis of TSC1, the cell lysates were collected and immunoprecipitated with IgG antibodies or TSC1 and protein A+G agarose beads (#P2012, Beyotime, Shanghai, China) at 4 °C. The mass spectrometry analysis was conducted by SpecAlly Life Technology Co., Ltd, Wuhan, China.

Sample preparation: the beads samples obtained from immunoprecipitation experiment were washed three times with pre-cooled PBS buffer to remove the remaining detergent. Then beads samples were incubated in the reaction buffer (1% SDC/100 mM Tris-HCl, pH 8.5/10 mM TCEP/40 mM CAA) at 95 °C for 10 min for protein denaturation, cysteine reduction and alkylation. The eluates were diluted with equal volume of H2O and subjected to trypsin digestion overnight by adding 1 μg of trypsin at 37 °C. The peptide was purified using self-made SDB desalting columns. The eluate was vacuum dried and stored at -20 °C for later use.

LC-MS/MS Detection: LC-MS/MS data acquisition was carried out on a Q Exactive HF-X mass spectrometer coupled with an Easy-nLC 1200 system (both Thermo Scientific). Peptides were first loaded onto a C18 trap column and then eluted into a C18 analytical column (75 μm × 250 mm, 3 μm particle size, 100 Å pore size, Acclaim PepMap C18 column, Thermo). Mobile phase A (0.1% formic acid) and mobile phase B (80% ACN, 0.1% formic acid) were used to establish a 120 min gradient. A constant flow rate was set at 300 nL/min. For DDA mode analysis, each scan cycle consisted of one full-scan mass spectrum (R = 120 K, AGC = 3e6, max IT = 50 ms, scan range = 350–1800 m/z) followed by 20 MS/MS events (R = 60 K, AGC = 2e5, max IT = 110 ms). HCD collision energy was set to 32. Isolation window for precusor selection was set to 1.6 Da. Former target ion exclusion was set for 40 s.

**GSEA for the key gene**

GSEA: KIRC patients were first divided into two groups according to the median expression level of the key gene. Then, differential expression analysis was applied between the high and low expression groups. Input genes for GSEA were sorted by their logFC values. Signaling pathways activated or suppressed by the key gene were decided by the normalized enrichment score (NES) value derived from GSEA.

ssGSEA: ssGSEA was used to calculate separate enrichment scores for each pairing of a KIRC sample and KEGG gene set. The ssGSEA score was further rescaled by min-max normalization method. Correlation analysis was performed between expression values of key gene and NES of signaling pathways.

**Plasmids and siRNA transfection and shRNA infection**

For transient transfection of siRNA and plasmids, cells were cultured in plates or dishes to undergo starvation treatment with 1 mL serum-free Opti-MEM medium (Gibco, USA) for 12 h. Then the indicated siRNA, plasmids and Lipofectamine 2000 (Thermo Fisher Scientific, China) were incubated together for 20 min in 1 mL serum-free Opti-MEM medium. The mixture was transferred to plates or dishes from centrifuge tubes. After the cells were transfected for 6 h, serum-free Opti-MEM medium was replaced with complete DMEM medium for another 72 h.

For shRNA infection, the 293T cells were cultured in plates or dishes to undergo starvation treatment with 1 mL serum-free Opti-MEM medium (Gibco, USA) for 12 h. Then the indicated shRNA, plasmids and Lipofectamine 2000 (Thermo Fisher Scientific, China) were incubated together for 20 min in 1 mL serum-free Opti-MEM medium. The mixture was transferred to plates or dishes from centrifuge tubes. After the cells were transfected for 6 h, serum-free Opti-MEM medium was replaced with complete DMEM medium supplemented with 1:100 sodium pyruvate. After 24 h, cell culture medium was collected and added to ccRCC cells with 12 ug/ml of polybrene. The cells with shRNA infection were selected with puromycin.

**RNA sequencing and analysis**

A total of 1 µg of RNA per sample was used as the starting material for RNA sequencing (RNA-seq). RNA integrity was assessed using the RNA Nano 6000 Assay Kit of the Bioanalyzer 2100 system (Agilent Technologies, CA, USA). Clean data (clean reads) were obtained by removing reads containing adapter, reads containing ploy-N and low-quality reads from raw data. At the same time, Q20, Q30 and GC content the clean data were calculated. All the downstream analyses were based on the clean data with high quality. Sequencing libraries were generated using the NEBNext Ultra RNA Library Prep Kit for Illumina (NEB, USA) following the manufacturer’s instructions, and index codes were added to attribute sequences to each sample. Clustering of the samples was performed on the cBot Cluster Generation System using the TruSeq PE Cluster Kit v3-cBot-HS (Illumina) according to the manufacturer’s instructions. After cluster generation, libraries were sequenced on an Illumina Novaseq platform, and 150-bp paired-end reads were generated. FeatureCounts v1.5.0-p3 was used to count the read numbers mapped to each gene. Differential expression analysis was performed using the DESeq2 R package (1.16.1), and the cluster Profiler R package was used to test the statistical enrichment of differentially expressed genes (DEGs) in KEGG (Kyoto Encyclopedia of Genes and Genomes) pathways. Three replicates were performed in each group.

The sequencing data was deposited in the GEO public dataset (GSE23816625).

**Table S1. The siRNA and shRNA sequences.**

| siCDK4 #1 | 5'- CACTCTTATCTACATAAGGATGA-3' |
| --- | --- |
| siCDK4 #2 | 5'- TACTAAAAATACAAAATTAGTCA-3' |
| shRNF26 #1 | 5′ -CCGGCACCGCGGAGTCTTGCTTTCATTGCTTCAAGAGAGCAAT GAAAGCAAGACTCCGCTTTTTG-3′ |
| shRNF26 #2 | 5′- CCGGCACCGCCGTGGTCCGGTTCACATGTTTCAAGAGAACATG TGAACCGGACCACGGCTTTTTG--3′ |
| shTSC1 #1 | 5'-CACCGTGGGAAACAGCATCTTCTTGATTCTCGAGAATCAAGAAGATGCTGTTTCCCA-3' |
| shTSC1 #1 | 5'-CACCGTGCGAATTCATCCGGAATTAGTCTCGAGACTAATTCCGGATGAATTCGCAC-3' |

**Table S2. The primer sequences for RT-qPCR.**

| Gene（Human） | Forward primer (5′ - 3′) | Reverse primer (5′ - 3′) |
| --- | --- | --- |
| GAPDH | ATGACAATGAATACGGCTACAGCA | GCAGCGAACTTTATTGATGGTATT |
| TSC1 | AACCTGTAGCACACGTCCTG | CGGCTTTGCCCACATATTCG |
| VEGFA | TCACCAAGGCCAGCACATAG | GAGGCTCCAGGGCATTAGAC |
